# Gene silencing of *Diaphorina citri* candidate effectors promotes changes in feeding behaviors

**DOI:** 10.1038/s41598-020-62856-5

**Published:** 2020-04-07

**Authors:** Inaiara de Souza Pacheco, Diogo Manzano Galdeano, Nathalie Kristine Prado Maluta, Joao Roberto Spotti Lopes, Marcos Antonio Machado

**Affiliations:** 1 Centro de Citricultura Sylvio Moreira, Instituto Agronômico de Campinas, Cordeirópolis, São Paulo, Brazil; 2 Universidade Estadual de Campinas, Campinas, São Paulo Brazil; 3 Instituto Agronômico de Campinas, Campinas, São Paulo Brazil; 4 Departamento de Entomologia e Acarologia, Escola Superior de Agricultura Luiz de Queiroz, Universidade de São Paulo, Piracicaba, São Paulo Brazil

**Keywords:** Entomology, RNAi

## Abstract

Insect effectors are mainly secreted by salivary glands, modulate plant physiology and favor the establishment and transmission of pathogens. Feeding is the principal vehicle of transmission of *Candidatus* Liberibacter asiaticus (*Ca*. Las) by the Asian citrus psyllid (ACP), *Diaphorina citri*. This study aimed to predict putative ACP effectors that may act on the Huanglongbing (HLB) pathosystem. Bioinformatics analysis led to the identification of 131 candidate effectors. Gene expression investigations were performed to select genes that were overexpressed in the ACP head and modulated by *Ca*. Las. To evaluate the actions of candidate effectors on *D. citri* feeding, six effectors were selected for gene silencing bioassays. Double-stranded RNAs (dsRNAs) of the target genes were delivered to *D. citri* adults via artificial diets for five days. RNAi silencing caused a reduction in the ACP lifespan and decreased the salivary sheath size and honeydew production. Moreover, after dsRNA delivery of the target genes using artificial diet, the feeding behaviors of the insects were evaluated on young leaves from citrus seedlings. These analyses proved that knockdown of *D. citri* effectors also interfered with ACP feeding abilities *in planta*, causing a decrease in honeydew production and reducing ACP survival. Electrical penetration graph (EPG) analysis confirmed the actions of the effectors on *D. citri* feeding behaviors. These results indicate that gene silencing of *D. citri* effectors may cause changes in *D. citri* feeding behaviors and could potentially be used for ACP control.

## Introduction

Insect pests are one of the main factors that reduce agricultural plant productivity. Global losses caused by these animals reach 220 billion dollars annually^1^. In addition to the damage caused by their actions, problems caused by insects can be increased by the transmission of several plant pathogens^2^.

Asian citrus psyllid (ACP), *Diaphorina citri* Kuwayama (Hemiptera: Liviidae), stands out as the most important agricultural pest among citrus crops^3^. ACP is a vector for the bacteria *Candidatus* Liberibacter asiaticus (*Ca*. Las), the causal agent of Huanglongbing (HLB). This disease affects all commercial citrus varieties by promoting plant decline and reducing fruit quality^4,5^. In the last fifteen years, HLB has spread quickly across the American continent and the citrus production in the USA declined 58%^4^. In Brazil, 34 million citrus trees have been removed since the first detection of HLB in 2004^6^.

So far, no efficient control for HLB has been found. Disease management consists of producing seedlings using *Ca* Las-free nursery stock, removal of infected trees and insecticide applications for vector control^7,8^. However, the extensive application of insecticides could select for ACP-resistant populations^9–11^.

*Diaphorina citri* acquire *Ca*. Las via a circulative-persistent manner during feeding^12,13^. The efficiency of *Ca*. Las transmission is affected by the duration of phloem ingestion by ACP^14^. Studies have been developed to understand the interactions of plants and ACP^15–17^. Nevertheless, this complex process remains unclear.

Additionally, salivary secretions have been described as important for insect-plant interactions^18–21^. These secretions may contain effector molecules that alter host plant physiology, activating or suppressing defense responses, consequently inhibiting or promoting plant infestation by the insects^22,23^. Insect effectors may also interfere with the transmission of microorganisms and plant-pathogen interactions as well^24–26^.

Many insect effectors have been described over the years as from saliva or salivary gland proteomic and transcriptomic data^27–31^. To unveil the actions of effector proteins on insect-hosts interactions, RNA interference (RNAi) has been applied. Knockdown of salivary effectors from *Nephotettix cincticeps* (Uhler, 1896) (Hemiptera: Cicadellidae), for example, reduced rice phloem ingestion by this insect^18^. Silencing of salivary mucin-like protein decreased the feeding performance and caused *Nilaparvata lugens* (Stål, 1854) (Hemiptera: Delphacidae) mortality through interactions with rice^32^. RNAi targeting whitefly, *Bemisia tabaci* (Genn.) (Hemiptera: Aleyrodidae), salivary laccase decreased the survival rates of this insect in tomato plants^19^.

Despite the existence of the ACP saliva proteome^33^, ACP effectors or salivary molecules that interact with host plants have not been identified until now^34^. In the present work, we predict secreted *D. citri* effectors using bioinformatics tools. Gene silencing through dsRNAs combined with an EPG technique was performed to verify the actions of the effectors on *D. citri* feeding behaviors.

## Results

### Candidate effector prediction

To generate a list of *D. citri* putative effectors, we used *Myzus persicae* Sulzer (Hemiptera: Aphididae) bioinformatics approach as a model for effector prediction^25^. With the intent of obtaining an accurate effectoromics dataset, proteins sequences used in these analyses were obtained from the following two distinct transcriptome and genomic sources: a transcriptome predicted from Arizona University studies^35^ and genomic sequences from the HLB consortium (citrusgreening.org).

Considering that insect effectors are secreted through the classical eukaryotic endoplasmic reticulum (ER)-Golgi pathway through salivary glands, the initial predicted *D. citri* secretome was composed of proteins presenting signal peptides and the absence of transmembrane domains. SignalP analysis allowed the identification of 2,099 proteins with signal peptides. Furthermore, screening using the TMHMM, Phorbius and GPI-SOM tools identified 670 proteins that contained transmembrane domains or GPI-anchor signals, and those proteins were removed from further analysis. Thereafter, the ACP secretome was composed of 1,443 proteins.

It has been reported that insect effectors are mainly species- or genus-specific^36^. Thus, we predicted conserved domains and identified characterized proteins through multiple alignments against public protein databases (Pfam and Uniprot). All proteins that showed ordinary domains were excluded. To select proteins that are only present in salivary glands, we also performed tBlast-X against the arthropod gut and salivary ESTs, removing proteins that may be secreted in the ACP gut. Thus, were identified 131 candidate effectors (Table S1) composed of 78% exclusively *D. citri* proteins and 22% showed similarity with arthropod saliva proteins (Table S2). Interestingly, many of the sequences showed intrinsically disordered regions (IDRs) (Table S1). These regions are abundant in phytopathogen effector proteins, interfere with protein secondary structure and allow protein folding in a stimulus-dependent manner^37^. The presence of enriched IDR proteins in the *D. citri* effectorome suggests a positive evolutionary selection of these proteins and potential functions through host interactions. Additionally, the subcellular localization of the predicted *D. citri* effectors suggests that the majority of the identified proteins act on the extracellular space (Table S1; Fig. S1). However, these proteins may also act on important host organelles such as the nucleus, chloroplast and mitochondrion (Table S1; Fig. S1).

### Selection of effectors candidates of *D. citri* by RT-qPCR

After the bioinformatic predictions, the screened candidate effectors were analyzed for their gene expression. Twelve putative effectors were evaluated (Tables 1 and S3) to identify genes that were expressed in different life stages, upregulated in the ACP head and showed expression modulated by *Ca*. Las.

**Table 1:** The twelve selected *D. citri* effectors for gene expression analysis.

Effector	ID	Characterization	Size (aa)	Subcellular localization
DCEF08	XP_008479196.1	WD repeat-containing protein 92	191	Extracellular space
DCEF10	XP_008482426.1	uncharacterized protein LOC103519088	228	Mitochondrion/Chloroplast
DCEF11	XP_026681229.1	embryonal Fyn-associated substrate-like	396	Mitochondrion/Chloroplast
DCEF19	DcWN_006647	uncharacterized protein	165	Extracellular space
DCEF22	DcWN_010436	uncharacterized protein	128	Extracellular space/Nucleus
DCEF23	DcWN_013243	uncharacterized protein	94	Extracellular space/Nucleus
DCEF26	DcWN_027964	uncharacterized protein	252	Mitochondrion
DCEF27	DcWN_028357	uncharacterized protein	259	Mitochondrion/Chloroplast
DCEF28	XP_026685929.1	uncharacterized LOC113471181	567	Nucleus
DCEF32	XP_008477481.1	uncharacterized protein LOC103514387	209	Golgi apparatus
DCEF33	XP_008475409.1	uncharacterized protein LOC103512422	197	Extracellular
DCEF35	XP_008468032.1	uncharacterized protein	142	Extracellular

First, the mRNA abundance of the candidate effector genes was evaluated in two different ACP life stages (nymph and adult). For both analyzed life stages, similar mRNA patterns were observed for the most candidates of the effectors (Fig. S2). DCEF22, DCEF28 and DCEF33 showed elevated mRNA levels in the nymph stage compared with the adult phase (Fig. S2).

**Figure 1:**
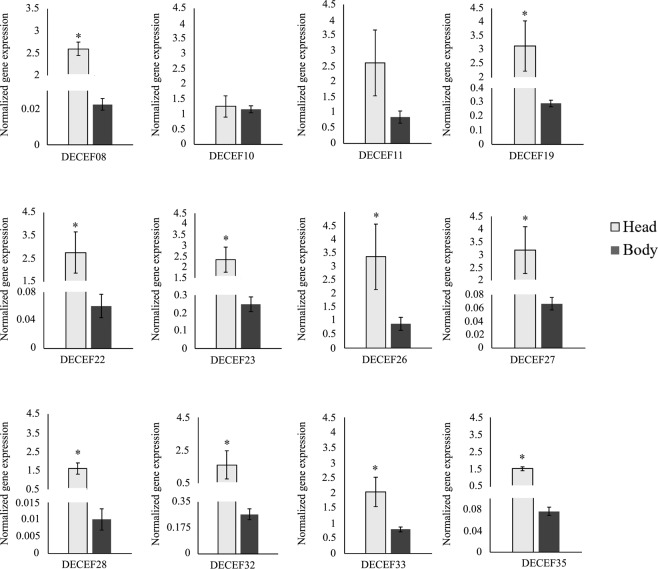
Relative gene expression of the 12 *D. citri* candidate effectors in the head and body. These data consist of normalized target mRNA levels to the mRNA abundance of the housekeeping genes S13 and S20. The results are the mean of three biological replicates per set. *corresponds to statistically significant differences amongst the treatments (t-test, P-value < 0.05).

Our goal was to select effectors that are present in the *D. citri* salivary gland or could be secreted by saliva. To achieve this aim, we evaluated the gene expression of candidate effectors from different body parts (head and body). The mRNA abundance of ten of twelve candidate effector genes was higher in the head than in the ACP body (Fig. 1). Candidate effectors that were not majorly expressed in the ACP head (DCEF10 and DCEF11) were excluded from further analysis.

**Figure 2:**
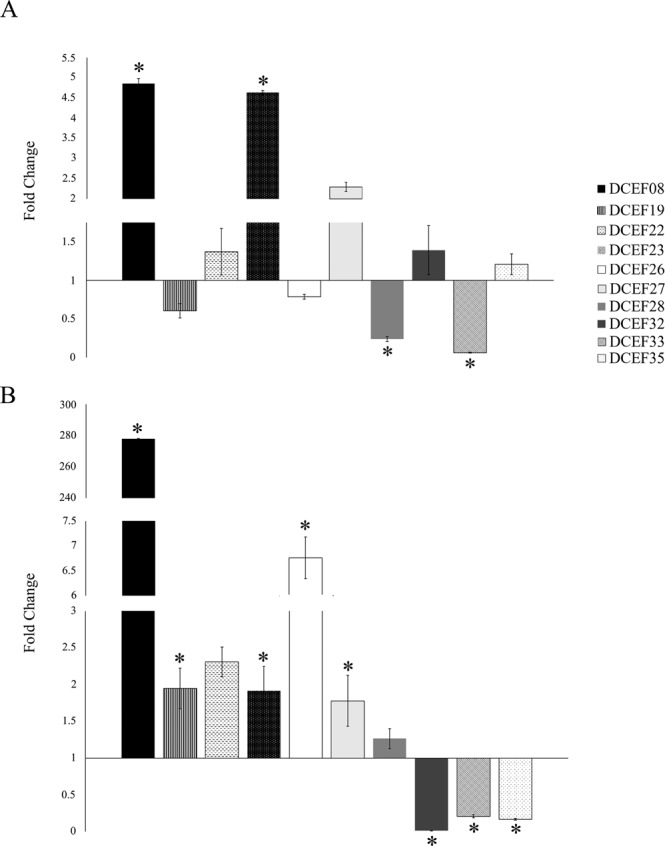
Relative gene expression of ten *D. citri* candidate effectors in nymph (**A**) and adult (**B**) *Ca*. Las-infected insects compared with *Ca*. Las-free nymph and adults. The results are the mean of three biological replicates per set. *corresponds to statistically significant differences amongst the healthy and *Ca*. Las-infected treatments (t-test, P-value < 0.05).

Furthermore, *Ca*. Las regulation of the gene expression of the ten candidate effectors was evaluated in nymphs and adults. Except for DCEF22, all the selected genes were significantly modulated by *Ca*. Las infection conditions in at least one of the analyzed life stages (Fig. 2). DCEF08 and DCEF23 were overexpressed in *Ca*. Las-infected insects compared with healthy insects in both the nymph and adult stages. The presence of *Ca*. Las contributes to increased DCEF19, DCEF26 and DCEF27 mRNA levels in ACP adults. DCEF33 was downregulated in both nymph and adult *Ca*. Las-infected ACPs. The DCEF32 and DCEF35 genes were downregulated in the *Ca* Las-infected adults. DCEF28 mRNA levels were reduced in *Ca* Las-infected nymphs compared with healthy nymphs (Fig. 2). Based on these results, the following six effectors were selected for the RNAi experiments: DCEF26, DCEF27, DCEF28, DCEF32, DCEF33 and DCEF35.

### Delivery of dsRNA by artificial diet

**Figure 3:**
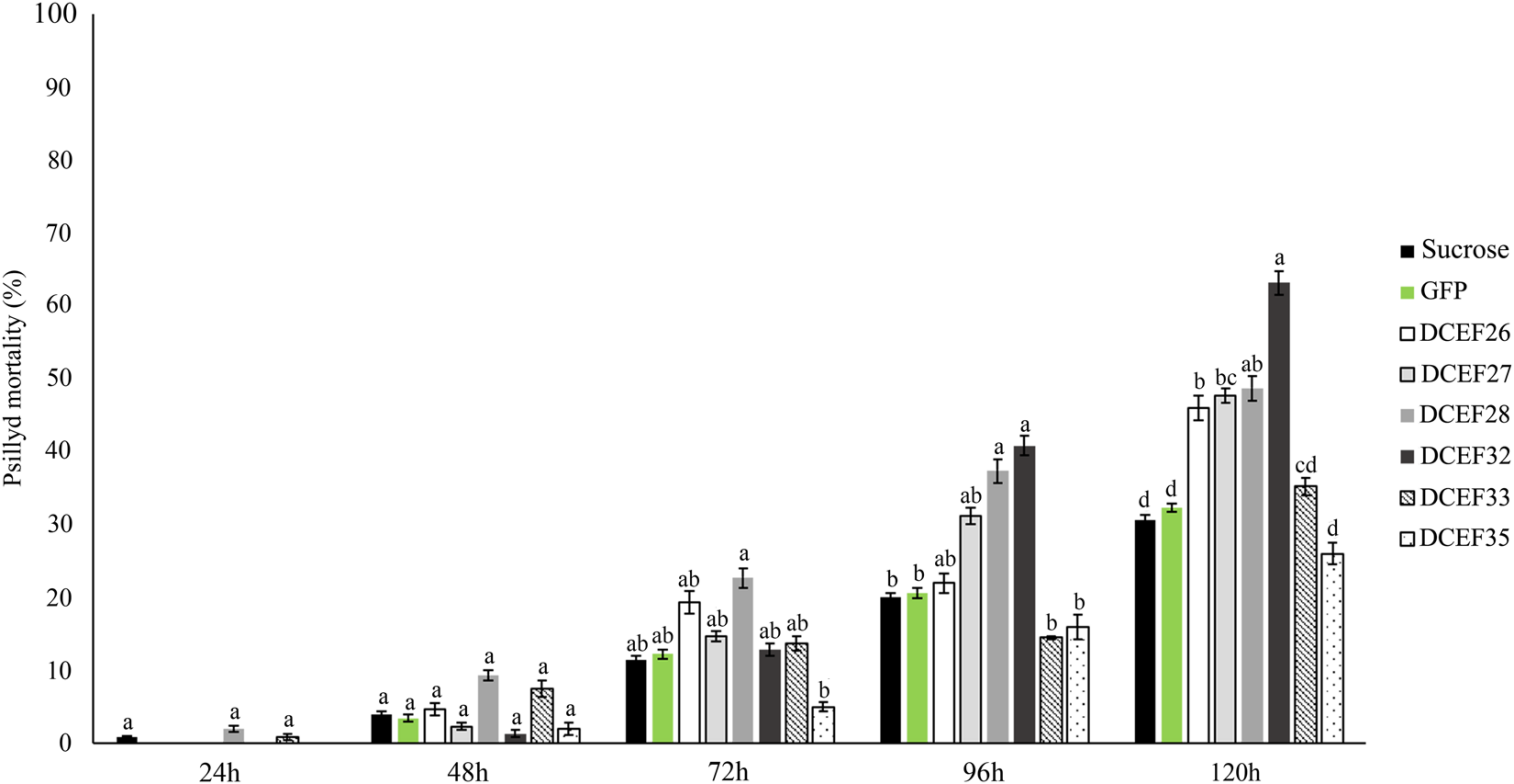
Mortality rates of *D. citri* fed an artificial diet containing 30% sucrose and 100 ng/µL of dsRNA for GFP or dsRNA for the effectors DCEF26, DCEF27, DCE28, DCEF32, DCEF33 or DCEF35. The mortality rates were evaluated daily during a 120-hour period. The data correspond to the average of three independent experiments. Different letters correspond to statistically significant differences amongst the treatments (Tukey test, P-value < 0.05).

DsRNA was delivered to ACP adults using a 30% sucrose-artificial diet with 100 ng/µL of dsRNA. After five days of continuous dsRNA delivery, we observed 46, 47 and 61% mortality for the DCEF26, DCEF27, and DCEF32 gene-fed insects, respectively (Fig. 3). DCEF28 treatment caused significant mortality during the first 72 hours of the experiment, reaching 48% mortality after five days. DCEF33 and DCEF35 showed fewer expressive effects on ACP mortality, causing 35 and 26% mortality, respectively (Fig. 3).

**Figure 4:**
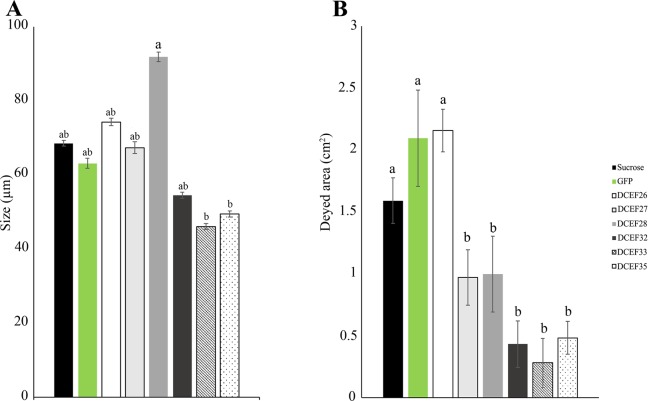
(**A**) Measurement (µm) of the ACP salivary sheath after treatment with dsRNAs for GFP, DCEF26, DCEF27, DCEF28, DCEF32, DCEF33 or DCEF35. (**B**) Honeydew tinted areas after treatment with dsRNAs for GFP, DCEF26, DCEF27, DCEF28, DCEF32, DCEF33 or DCEF35. The data correspond to the average of three independent experiments. Different letters correspond to statistically significant differences amongst the treatments (Tukey test, P-value < 0.05).

To verify the interference of silencing the effectors on ACP feeding, salivary sheath evaluation was performed through microscopy analysis. No structural differences in the salivary sheaths were observed between the treatments and control (Fig. S3). However, the salivary sheath sizes of *dsDCEF32*-, *dsDCEF33*- and *dsDCEF35*-treated insects were 20, 32 and 27% lower than the sucrose control, respectively. *dsDCEF*28-treated insects exhibited a 45% increase in salivary sheath size compared with the control (Fig. 4).

Moreover, the effects of effector silencing on ACP ingestion abilities were evaluated by quantification of the excretions deposited on filter paper at the bottom of the cages. A significant reduction in honeydew was observed in five of six treatments (Fig. 4). The DCEF32, DCEF33 and DCEF35 treatments caused the strongest reduction in honeydew production, presenting tinted areas that were 72, 82 and 69% lower than the sucrose control, respectively. A 38 and 37% reduction in the tinted area was observed for the DCEF27 and DCEF28 treatments, respectively. No differences in honeydew production were observed between the DCEF26 treatment and control. These results corroborate the hypothesis that the silencing of selected candidate effectors may affect *D. citri* feed performance.

**Figure 5:**
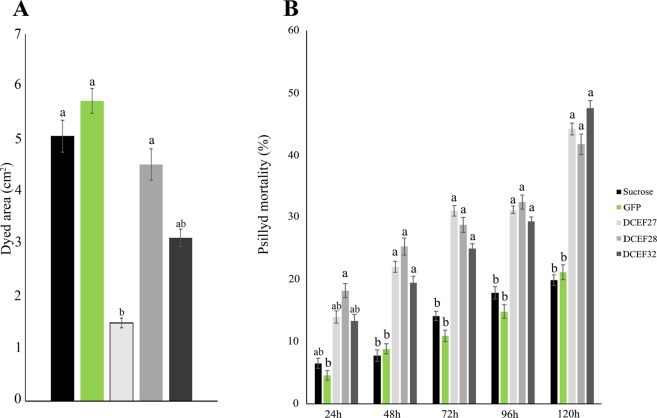
(**A**) Honeydew tinted area from DCEF27-, DCEF28-, and DCEF32- silenced ACPs after feeding with ‘Rangpur lime’ leaves. (**B**) Mortality rates of *D. citri* on Rangpur lime leaves after treatment with dsRNAs for the DCEF27, DCE28, or DCEF32 effectors. ACPs were treated with effector dsRNAs *in vitro* and then transferred to the citrus plants for feeding. The mortality rates were evaluated daily during a 120-hour period. The data correspond to the average of four independent experiments. Different letters correspond to statistically significant differences amongst the treatments (Tukey test, P-value < 0.05).

Compared with *in vitro* conditions, plant responses could alter insect feeding performance^31^. To confirm the silencing interference on ACP feeding *in planta*, we evaluated the ACP behaviors on citrus plants after delivery of dsRNAs. This analysis was performed using the following three candidate gene effectors: DCEF27, DCEF28 and DCEF32. ACP adults were treated with dsRNA via artificial diet for five days. Then, insects were transferred to ‘Rangpur lime’ seedling leaves for five days. Mortality was evaluated daily and the quantification of honeydew droplets and gene expression analysis were performed at the end of the assays. After the first 48 hours, all treatments showed elevated mortality compared with the sucrose and GFP controls (Fig. 5), presenting 44, 41 and 47% mortality for the DCEF27, DCEF28 and DCEF32 gene treatments after five days, respectively. The honeydew analysis demonstrated a reduction in excreta production by dsRNA-treated insects (Fig. 5). A decrease of 10 and 38% for the tinted area was observed for the DCEF28 and DCEF32 treatments, respectively, compared with sucrose. *dsDCEF27-*treated insects showed the strongest reduction in honeydew production, showing tinted areas that were 70% lower than the controls (Fig. 5). These results demonstrated that knockdown of *D. citri* effectors could also interfere with ACP feeding *in planta*.

### Evaluation of *D. citri* feeding behaviors

**Figure 6:**
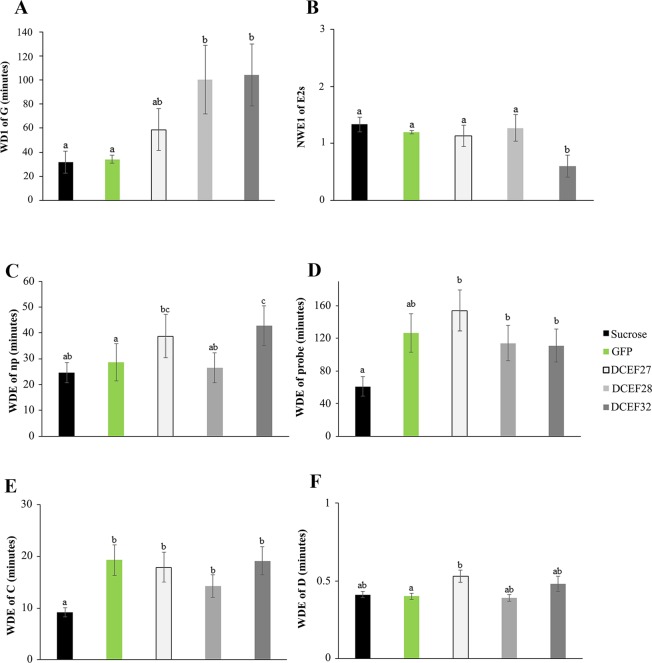
Comparison of ACP waveforms of feeding behaviors after *in vitro* delivery of dsRNAs for the DCEF27, DCEF28, or DCEF32 effectors and the sucrose and GFP dsRNA controls during an 8-h recording on the ‘Rangpur lime’ seedlings. The waveform duration per insect (WDI) of xylem (**A**). The number of waveform events per insect (NWEI) for E2s (**B**). The waveform duration (min) per event (WDE) for non-probe (np) (**C**), probe (**D**), waveform C (**E**), and waveform D (**F**). Different letters indicate statistically significant differences according to the Tukey test (for Gaussian variables) or Kruskal-Wallis test (for non-Gaussian variables) (P-value < 0.05).

To confirm the changes to *D. citri* feeding behaviors caused by the silencing of candidate effectors, EPG analysis was applied to reveal the differences by the treatments. EPG recordings were performed on adult females after dsRNA delivery via an artificial diet for five days. The DCEF28 and DCEF32 treatments showed long feeding periods on xylem (waveform G) (Fig. 6A) compared with the controls (sucrose and GFP). Moreover, the proportion of individuals that produced waveform G (PPW) was lower for the treatment GFP than in the other treatments (Table 2).

**Table 2:** Proportion of *Diaphorina citri* individuals that produced a specific waveform type (PPW) on ‘Rangpur lime’ seedlings during a 10-h recording.

Waveform*	PPW							*P*
	Sucrose	GFP	DCEF27	DCEF28	DCEF32	*X*^2^	df	
G	9/15 a	6/15 b	11/15 a	13/15 a	12/15 a	9.44	4	0.049
D	15/15 a	15/15 a	13/15 a	12/15 a	11/15 a	8.08	4	0.089
E1	15/15 a	15/15 a	13/15 a	12/15 a	11/15 a	8.08	4	0.089
E2	15/15 a	15/15 a	12/15 a	12/15 a	8/15 b	15.45	4	0.004
E2 > 10 min	15/15 a	15/15 a	12/15 a	12/15 a	7/15 b	18.79	4	<0.001

*Waveforms: (G) active intake of xylem sap, (D) first contact with phloem, (E1) salivation in the phloem sieve tubes, and (E2) phloem sap ingestion^54^. Proportions followed by the same letter in the same row do not differ significantly (P-value > 0.05) using chi-square (*X*^2^) test for pairwise comparisons.

Sustained phloem ingestion (E2 > 10 minutes) was significantly reduced for the DCEF32 treatment compared to the other treatments (Fig. 6B). Furthermore, the proportion of individuals that underwent ingestion in the phloem (E2 and E2s) was lower for the DCEF32 treatment when compared to the other treatments (Table 2).

The mean duration per event (WDE) of non-probing was significantly longer for the DCEF32 treatment than for the sucrose and GFP treatments (Fig. 6C). The WDE of C (stylet pathway) and probe were significantly shorter for the sucrose treatment control compared with the other treatments (Fig. 6D,E). The WDE of D was significantly longer for the DCEF27 treatment when compared with the GFP treatment (Fig. 6F).

### Gene expression analysis

**Figure 7:**
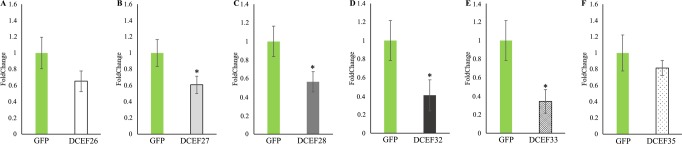
Relative gene expression of the candidate effectors DCEF26 (**A**), DCEF27 (**B**), DCEF28 (**C**), DCEF32 (**D**), DCEF33 (**E**) and DCEF35 (**F**) compared with the GFP control. Gene expression evaluation occurred on ACP adults after feeding with an artificial diet containing dsRNAs for the effectors or dsRNAs for GFP for five days. The data correspond to the mean of three independent experiments. *corresponds to statistically significant differences amongst the treatments (t-test, P-value < 0.05).

After five days of dsRNA delivery via an artificial diet, gene expression analysis showed a reduction in the mRNA levels of all the effector genes, confirming the knockdown of the candidate effectors (Fig. 7). The strongest mRNA reduction was observed for the DCEF33 and DCEF32 genes, which presented 63 and 59% inhibition in their mRNA levels, respectively. The DCEF26, DCEF27 and DCEF28 genes showed a reduction of 34, 39 and 43% of their mRNA levels, respectively, compared with the GFP control. The DCEF35 effector presented an 18% inhibition in gene expression (Fig. 7).

**Figure 8:**
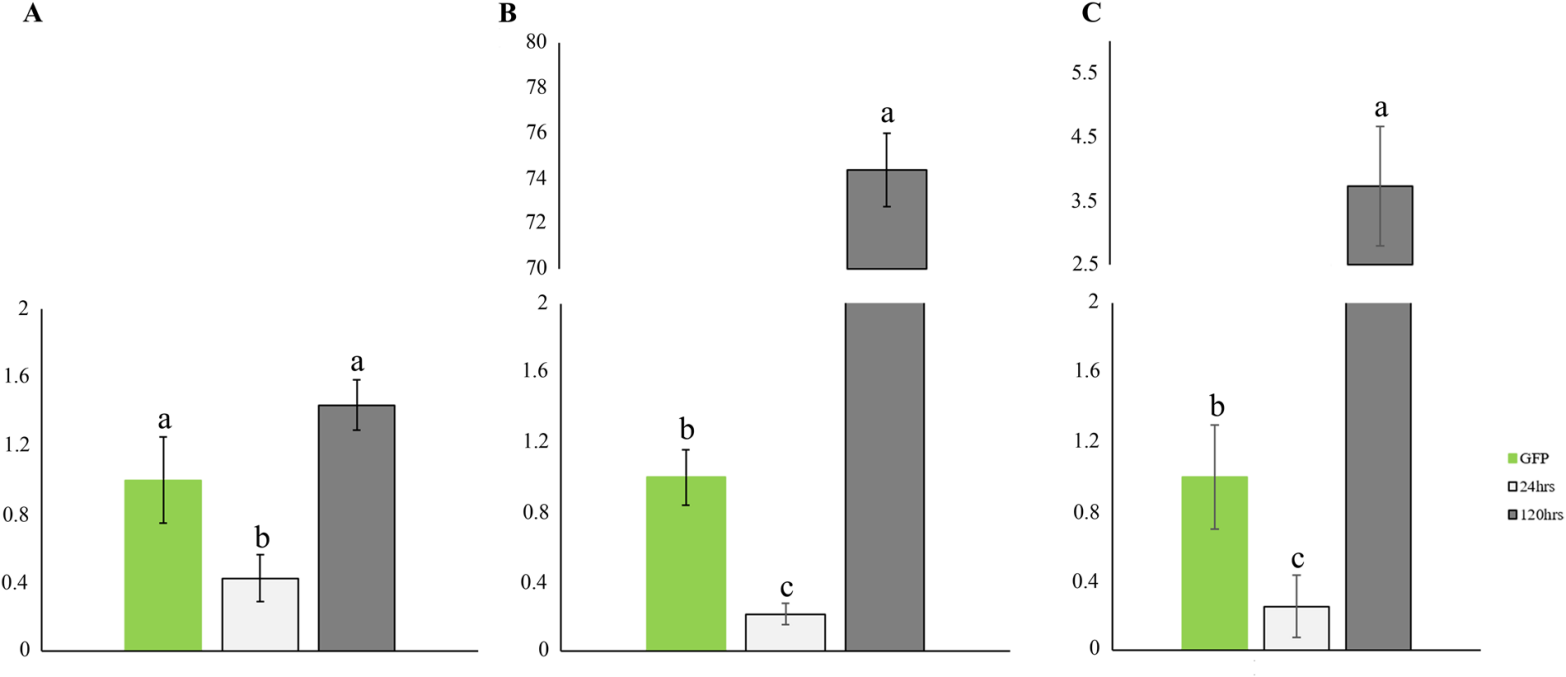
Relative gene expression of the candidate effectors DCEF27 (**A**), DCEF 28 (**B**), and DCEF32 (**C**) compared with GFP control observed 24 hours (light grey) and 120 hours (dark gray) after ACP adults were treated with dsRNAs for effectors and transferred for feeding on ‘Rangpur lime’ leaves. The data correspond to the average of four independent experiments. Different letters correspond to statistically significant differences amongst the treatments (Tukey test, P-value < 0.05).

To confirm the maintenance of effector silencing in the *in planta* experiments, gene expression analysis was performed 24 and 120 hours after the transfer of the dsRNA-treated insects from an artificial diet to ‘Rangpur lime’ plants (Fig. 8). During the first 24 hours when the dsRNA-treated ACPs were fed plants, all the analyzed genes showed a reduction in their mRNA levels, proving that the *D citri* effectors still silenced (Fig. 8). However, the dsRNA-treated *D. citri* showed restoration of effector gene mRNA levels after 120 hours of feeding with citrus plants (Fig. 8).

## Discussion

### Candidate effectors prediction and validation

Salivary proteins are important for proper insect feeding and promoting lubrification, digestion and penetration^38^. These proteins also play important roles in plant-insect interactions^34^. For example, the potato aphid *Macrosiphum euphorbiae* Thoma (Hemiptera: Aphididae) presents an effector that interacts with two plant defense compounds to enhancing aphid fecundity^39^. The Hessian fly, *Mayetiola destructor* (Say 1817) (Diptera: Cecidomyiidae), phosphatase 2 C effector family elicits effector-triggered immunity in wheat by interacting with signal transduction pathways^40^. Furthermore, feeding is the principal vehicle of *Ca*. Las acquisition by ACP. Studies have demonstrated that long phloem ingestion by ACP nymph instars is responsible for the high acquisition efficiency of *Ca*. Las by immature *D. citri* instars^14^. Based on this, we proposed the identification of *D. citri* candidate effectors, looking for proteins important for interactions between ACP, citrus and *Ca*. Las.

Bioinformatics tools are the first line for the prediction of prokaryote or eukaryote effectors^41,42^. Independent of species, effector prediction is based on the following same criteria: amino acid composition and primary protein sequences, such as the presence of signal peptides, absence of anchor membrane domains, and secondary characteristics such as species-specific sequences, nuclear localization signals (NLSs), three-dimensional structure, positive selection, etc^41^. Thus, we use a Pea aphid bioinformatics pipeline^25^ as a model to predict ACP candidate effectors. Pea aphid effectors are some of the best characterized insect effectors^28,43,44^, and these tools allowed the identification of 131 ACP candidate effectors (Table S1). Saliva or salivary gland protein sequences are very often applied for the prediction of insect effectors^45–47^. However, in the absence of this kind of sequence source, the Pea aphid pipeline was successfully applied for mite effector prediction using full body genomics and transcriptomic data^48^, as was also applied in this work. These studies support the legitimacy of the results obtained here. Seeking to ensure that the predicted candidate effectors are manly secreted in ACP saliva, all proteins that presented similarity with arthropod gut proteins were excluded. We identified twenty-nine *D. citri* candidate effectors showing similarity with arthropod salivary proteins (Table S2), supporting the hypothesis that these effectors may be produced and secreted by *D. citri* salivary glands.

The major ACP candidate effectors are composed of *D. citri-*unique unknown function proteins (Table S1). Prediction of aphid effectors also showed a large number of unknown functional proteins^28,29^. Insect effectors are mostly species- or genus-specific^36,43,49^. An elevated number of unique uncharacterized proteins may indicate that the effectors are highly host-specific, resulting from strong selection pressures to adaptation between insects and their host plant^28,29^. Bioinformatics analysis revealed nuclear localization signals (NLSs) and chloroplast transit peptides (cTP) in several ACP effectors (Table S1; Fig. S1), showing that these putative effectors may act on important host organelles (Table S1; Fig. S1). The VPS52 effector protein from *M. persicae* is relocated to post-Golgi and pre-vacuolar compartments in *Nicotiana benthamiana* plants and interferes with aphid virulence^50^. The *B. tabaci* effector Bsp9 acts on the host cell cytoplasm, interacting with plant WRKY33 transcription factors to suppress defenses^51^.

*D. citri* effectors genes are expressed during different ACP life stages (Fig. S2). These candidate effectors genes are also overexpressed in *D. citri* head compared with their body, supporting the hypothesis that these proteins may be secreted by ACP saliva (Fig. 1). Over-expression in the insect head was also a criterion applied to discriminate effectors from aphid and mite species^29,48^. Moreover, gene expression analysis of *Ca*. Las-infected and *Ca*. Las-free nymphs and adults showed that the *D. citri* effectors genes are modulated in response to these bacteria (Fig. 2), which is indicative that these genes could interfere with the *Ca*. Las transmission process by *D. citri*.

### Silencing of candidate effectors on *D. citri* feeding behavior

Effectors are strongly related to insect feed performance. Salivary compounds may elicit or suppress plant defenses, increasing or inhibiting insect feeding on its host plants^52^. Thereby, feeding performance and fecundity are the main phenotypes evaluated in insect effector studies. Silencing of angiotensin-converting enzymes (ACEs) genes from *A. pisium* interferes with phloem sap ingestion, enhancing the aphid mortality^23^. The knockdown of salivary endo-b-1,4-glucanase from *N. lugens* decreased its feeding performance in rice^53^. To ensure that the predicted *D. citri* effectors are feeding-related, we evaluated the ACP feeding performance after gene silencing.

Classical strategies for evaluating insect feeding include monitoring the salivary sheath and excreta production^54,55^. We observed that knockdown of ACP effectors *in vitro* assays promoted changes in the salivary sheath size and a strong reduction in honeydew production (Fig. 4), which indicates that the size of the salivary sheath may affect ACP ingestion.

The salivary sheath is one of two common salivary secretions produced by phytophagous hemipterans. Resulting from the solidification of insect gelling saliva, the salivary sheath is a solid structure that covers insect stylet providing orientation and stability during the piercing process and also protects the insect stylet from plant defenses^56–58^. The production of this structure is tightly related to appropriate insect feeding. Even so, excretion production is directly associated with insect ingestion^59,60^. Similar phenotypes were observed upon knockdown of salivary sheath proteins in the brown planthopper *N. lugens*, which also resulted in a reduction of honeydew amounts^61^.

However, these alterations did not directly affect mortality rates. The DCEF33 and DCEF35 treatments, which showed the strongest reduction in salivary sheath size and honeydew droplets, presented no significant mortality in the artificial diet experiments (Fig. 3). Similar patterns were observed when silencing the *NlShp* gene from *N. lugens*, which cause changes in insect feeding behaviors *in vitro*; however, knockdown of this gene did not cause significant insect mortality^31^.

Additionally, knockdown of ACP effectors also interferes with ACP feeding *in planta*. An expressive decrease in excreta production in *dsDCEF27* and *dsDCEF32-*treated insects and elevated mortality were observed when these insects were fed the ‘Rangpur lime’ leaves (Fig. 5).

Interference of feeding performance caused by effector silencing was proven by evaluation of *D. citri* feeding behaviors through EPG analysis, which showed a significant reduction in phloem ingestion by the *dsDCEF32*-treated insects. Only 50% of the *dsDCEF32*-treated insects showed E2 and E2 > 10 min waveforms (Table 2). Moreover, the number (NWEI) of E2 > 10 min waveforms was reduced by 50% for the DCEF32 treatment compared with the other treatments (Fig. 6B). These results indicate that *dsDCEF32*-treated insects seldomly performed passive phloem ingestion. Since *Ca*. Las is a phloem-inhabiting bacteria^62^, these results allow the hypothesis that DCEF32 may be important for the *Ca*. Las transmission process. Luo and collaborators (2015) showed that ACP adults that displayed an E2 waveform (passive phloem-sap uptake from sieve elements) were subsequently detected as *Ca*. Las-positive, demonstrating these EPG waveforms are strongly associated with *Ca*. Las acquisition^63^. In contrast, the strong correlation between the duration of phloem salivation (E1 waveform) and *Ca*. Las inoculation was described by Wu and collaborators (2016). These studies demonstrated that phloem phases (E1 and E2) are a critical point for the transmission of *Ca*. Las by *D. citri*^63–65^.

An interesting observation was the increased frequency and time of waveform G, which represents xylem ingestion, for the DCEF28 and DCEF32 treatments (Fig. 6A). In general, the G waveform presents low frequency and duration during *D. citri* feeding behaviors. Previous analysis demonstrated that the G waveform occurs on approximately 30% EGP recordings for *D. citri* adults, with durations ranging from 9 to 18% of the recording time^14,66,67^. Surprisingly, our results showed a proportion of individuals that had G waveforms (PPW) greater than 70% for the dsRNA-effector treatments (Table 2). The *dsDCEF28*- and *dsDCEF32*-treated insects showed longer feeding periods on xylem vessels (Fig. 6A; Table S4). G waveform formation for phloem sap feeders is frequently associated with dehydration or osmotic balance^68,69^. During citrus-ACP interactions, an increase in the G waveform duration was reported when ACP feeds on CLas-infected plants^14,64^. *D. citri* that performed xylem ingestion more often and for long periods were also observed on mature leaves compared with young ones due to the more pronounced sclerenchymatous ring around phloematic vessels, which difficult for phloem ingestion^67^. In this case, we hypothesize that xylem ingestion occurs to compensate for inefficient phloem ingestion^67^.

### Effect of silencing on candidate effectors gene expression

Knockdown of *D. citri* effectors was proved by the significant reduction of the mRNA levels of these genes after delivery of dsRNA via an artificial diet and 24 hours after transferring the dsRNA-treated insects to Rangpur plants (Figs. 7 and 8). Nevertheless, after 120 hours of constant feeding of dsRNA-treated insects with citrus plants, the mRNA levels of *D. citri* effector genes were restored (Fig. 8), demonstrating that the silencing effects of the analyzed genes are transient in the absence of a constant dsRNA source. The lack of an efficient silencing signal amplification system and the presence of nucleases that degrade dsRNAs may be factors that could explain the observed phenomena, though none of these observations have been previously described for ACP^70^. Several hemipterans species have been reported to have nucleases that quickly degrade dsRNAs in different body fluids^71^. Differences in nuclease activity in different tissues were also described as an important factor for determining dsRNA delivery strategies for some insects^72–74^.

## Conclusion

Most of the RNAi studies applied to *D. citri* control are focused on identifying candidate genes that promote significant mortality or insecticide susceptibility as well as new efficient strategies for dsRNA delivery, however, no practical use of this technology against *D. citri* has emerged. Studies focused on *D. citri-*Citrus- *Ca*. Las are recent, and many gaps regarding the genetic and molecular aspects of these interactions need to be fulfilled. Thus, the increase of the knowledge about *D. citri* biology and its interactions with citrus and *Ca*. Las is essential to develop new and efficient control strategies for HLB. Despite of recent identification of the *D. citri* saliva proteome, ACP effectors or salivary molecules that interact with plant hosts have not been identified to date. This study predicted *D. citri* effectors using a classical bioinformatics pipeline. Gene expression analysis combined with RNAi and EPG techniques allowed us to prove that the predicted effectors are directly related to ACP feeding performance, which is similar to the majority of insect effectors identified to date. The DCEF32 gene stands out amongst the identified effectors due to its action on *D. citri* phloem ingestion, which is the main colonization site of *Ca*. Las in plants. Moreover, gene expression analysis showed that the DCEF32 gene is modulated in response to *Ca*. Las during both the nymph and adult stages. These results may indicate that the DCEF32 effector could participate in *Ca*. Las-*D. citri* interactions. However, how *D. citri* effectors interact with host plants and/or *Ca*. Las still needs to be investigated. Understanding the participation of *D. citri* effectors in the HLB pathosystem can elucidate the molecular mechanisms of *Ca*. Las transmission by *D. citri* and its interaction with citrus plants. This knowledge is essential to develop novel and efficient control strategies for HLB.

## Materials and Methods

### Sequence database

Transcripts sequences from Arizona University transcriptome studies^35^ were initially used as a database. Complementary version 0.9 of the transcriptome and version 2.0 of the *D. citri* genome available on Citrus greening Solutions website (citrusgreening.org) were added to the bioinformatics analysis.

### Bioinformatics analysis

The bioinformatics for the identification of *D. citri* candidate effectors was based on a pipeline for the prediction of aphid effectors^25^. First, redundant sequences and proteins with 1000 > amino acids were removed from the analysis. Redundant sequences were those that presented a similarity > 95% and an e-value < 10^−10^. Secreted proteins were predicted using SignalP 4.0^75^. Proteins that had transmembrane domains were predicted using the TMHMM and GPI-SOM software^76,77^ and removed from future analysis. InterPro, Uniprot and Prosite^78,79^ were used to identify and exclude ordinary proteins. Proteins that displayed similarity with arthropod gut proteins were removed through blastX against arthropod gut ESTs. To identify potential *D. citri* salivary proteins, blastX against arthropod salivary proteins was performed. Additionally, prediction of ACP candidate effector subcellular localization was performed using the NetNES 1.1 Server, Localizer and LocTree tools^80–82^.

### Asian citrus psyllid colony

Mesh cages containing *Murraya paniculata* were used to rear healthy *D. citri* at 25 ± 2 °C under a 14:10 h (light:dark) photoperiod and 60 to 70% relative humidity (RH). CLas-infected ACP colonies were reared on *Ca*. Las-infected Pera sweet orange trees (symptomatic and PCR positive) were grown under greenhouse conditions for light, temperature and humidity. Both ACP populations were maintained at the biotechnology laboratory at the Centro de Citricultura ‘Sylvio Moreira’.

### Nuclei acid isolation and cDNA synthesis

RNA extraction was performed using a Direct-zol RNA MiniPrep and DNAse I Set (Zymo Research, Irvine, CA, USA) according to the manufacturers’ instructions. A NanoDrop ND 8000 spectrophotometer (NanoDrop Technologies, Wilmington, DE, USA) was used to measure the RNA integrity and quality. One microgram of extracted RNA was applied for cDNA synthesis using the GoScript reverse transcription system (Promega) according to the manufacturer’s protocol. After the reaction, the cDNA was diluted 1:25 for further analysis.

### Double-strand RNA synthesis

The target sequences of ACP candidate effectors were amplified by RT-PCR using specific gene primers conjugated with 19 bases of the T7 RNA polymerase promoter (Table S3). The GFP sequence was amplified from the pIG1783f plasmid^83^. The PCR reaction was performed using 5 μL of cDNA, 25 μL of Gotaq colorless MasterMix (Promega), and 100 nM of each primer pair in a final volume of 50 μL. Thermal profiles were determined using 10 cycles of 94 °C for 30 s, 1 min at 10 °C above the optimal primer annealing temperature, and 72 °C for 1.3 min, followed by 35 cycles of 94 °C for 30 s, 1 min at the optimal primer annealing temperature, and 72 °C for 40 s.

DsRNAs were synthesized from purified PCR products using the MEGAscript RNAi kit (Ambion, catalog no. AM1626) according to the manufacturer’s protocol. The purified dsRNAs were quantified spectrophotometrically at 260/280 nm and the integrity was examined by agarose gel electrophoresis.

### Delivery of dsRNA and bioassay *in vitro*

Ten-day-old adults were used for the RNAi experiments. The insects were maintained in plastic cages containing a sachet composed of 100 µL of an artificial diet (30% of sucrose and 0.1% green and 0.4% yellow food dyes) homogeneously distributed between two parafilm layers^84^ and filter paper on the bottom of the cage. Feeding assays were performed for a period of five days at room temperature under a 14:10 h (light: dark) photoperiod and 60 to 70% HR. Each treatment consisted of five biological replicates (10 insects per replicate). Insect mortality was evaluated daily. Three independent bioassays were performed.

After the feeding period, live insects were collected for further gene expression analysis. Salivary sheaths present on the parafilm membrane were dyed using 0.1% safranin, quantified and photographed with a light microscope using a 40x objective. The sizes of the salivary sheaths were measured using the ImageJ software^85^. Honeydew droplets present on the filter paper were dyed with 2% ninhydrin, counted and measured using the Quant software^86^. The remaining artificial diet was collected and examined by electrophoretic roll to observe the dsRNA stability (Fig. S4). Statistical analyses were performed using one-way ANOVA (P < 0.05) with the GraphPad Prism software^87^.

### *In vivo* bioassay for silencing effect evaluation

After the *in vitro* dsRNA delivery described above, living insects were confined to ‘Rangpur lime’ seedling leaves using clip cages. The clip cages were made from 90-mm in diameter plastic Petri dishes. A hole (7 mm) was made on the lateral side of the Petri dishes to facilitate the accommodation of a leaf blade and petiole. The leaf was attached to the Petri lid using adhesive tape. The filter paper was placed in the bottom of the clip cage to collect the honeydew droplets. The plates were sealed using a cotton and PVC film. Mortality was evaluated for five days and the feeding abilities were analyzed based on the quantity of honeydew droplets present on filter paper as described before. The experiment was repeated four times using four biological replicates for each treatment (ten adults ACP per biological replicate). Statistical analyses were performed using one-way ANOVA (Tukey test) and the t-test (P < 0.05) using the GraphPad Prism software^87^.

### Feeding behavior evaluation of *D. citri* by electron penetration graph (EPG)

After delivery of the dsRNA via the artificial diet as described above, we performed feeding behavior assays using an EPG technique, which allows for the real-time study of an insect’s stylet activities in plant tissues. Females ACPs were anesthetized for 5 seconds with CO_2_ and immobilized using a vacuum chamber under a dissecting microscope. Then, a gold wire (3 cm length, 20 µm in diameter; EPG Systems, Wageningen, The Netherlands) was attached to the ACP pronotum with a small droplet of water-based silver glue. The opposite end of the gold wire was glued to a thin copper wire (2 cm length), which was connected to the EPG probe. Another copper electrode (10 cm long, 2 mm wide) was inserted into the plant soil.

The insects were placed on the abaxial surface of a young ‘Rangpur lime’ leaf. The EPG waveforms were recorded using a Direct Current 8-channel EPG device model Giga-8d, with Stylet+ for Windows software (EPG Systems). The recordings were carried out in a room at 25 ± 1 °C inside a Faraday cage (for electrical noise isolation) for 8 hours. The waveforms recorded for *D. citri* feeding behaviors were characterized according to previous reports^54^. The output is given in an Excel workbook^88^ to calculate the treatment mean for each EPG variable. Fifteen individual recordings were performed for each treatment.

The EPG waveforms previously described for *D. citri*^66^ were identified as follows: waveform np (non-probing behavior); waveform C (which indicates movement of the stylets in the intercellular apoplastic space); waveform D (a short waveform always observed between waveforms C and E1); E1 waveform (indicates salivation into phloem sieve elements); waveform E2 (correlated with passive phloem-sap uptake from sieve elements); and waveform G (active intake of xylem sap).

The experimental design was entirely randomized. The EPG data were transformed when necessary with ln (x + 1) or √(x + 1) to reduce the heteroscedasticity and improve the normal distribution. All parameters were analyzed with Tukey’s test (P < 0.05). If the data did not follow a normal distribution according to the Shapiro-Wilk normality test, a nonparametric Kruskall-Wallis test (P < 0.05) was performed. The proportion of individuals that produced a specific waveform type (PPW) was compared among the different treatment groups using the chi-square test. All data were analyzed using the IBM Statistics SPSS 22.0 software^89^.

### Gene expression analysis

To select candidate effectors by RT-qPCR, pools of 10 adults and 50 nymphs were employed for RNA extraction as described before. Gene expression analysis of different body parts was conducted using 10 heads and 10 bodies from ACP adults. The measurement of gene expression in insects silenced by dsRNA was performed using RNA extracted from a pool of four ACP adults per biological replicate from both the artificial diet and plant experiments.

RT-qPCR reactions were performed using 6.5 µL of GoTaq qPCR MasterMix (Promega), 120 nM of each gene-specific primer pair and 3 μL of the diluted cDNA in a final volume of 12.5 μL. The amplification cycles were performed on a 7500 Fast Real-Time PCR System device (Thermo Scientific, Waltham, MA, USA) using the following standard thermal profile: 95 °C for 20 s followed by 40 cycles of 95 °C for 3 s and 60 °C for 30 s. Five technical replicates were analyzed for each sample. The Cq values and the primer efficiency were estimated using the Miner software (http://miner.ewindup.info). The normalized gene expression analyses were performed with the 2^ΔCq^ method and relative gene expression was estimated using the 2-^ΔΔCq^ method using the ribosomal genes S20 and S13 as reference genes. For statistical analysis, one-way ANOVA (Tukey test) and the t-test (P < 0.05) were performed using the GraphPad Prism software^87,90^.

## Supplementary information

supplementary information.

supplementary information 2.

supplementary information 3.

supplementary information 4.

supplementary information 5.

supplementary information 6.

supplementary information 7.

supplementary information 8.
