# Supplementary figures and images for: Gene silencing of Diaphorina citri candidate effectors promotes changes in feeding behaviors
Source: Sci Rep. 2020 Apr 7;10:5992. doi: 10.1038/s41598-020-62856-5 (PMC7138822; doi:10.1038/s41598-020-62856-5)

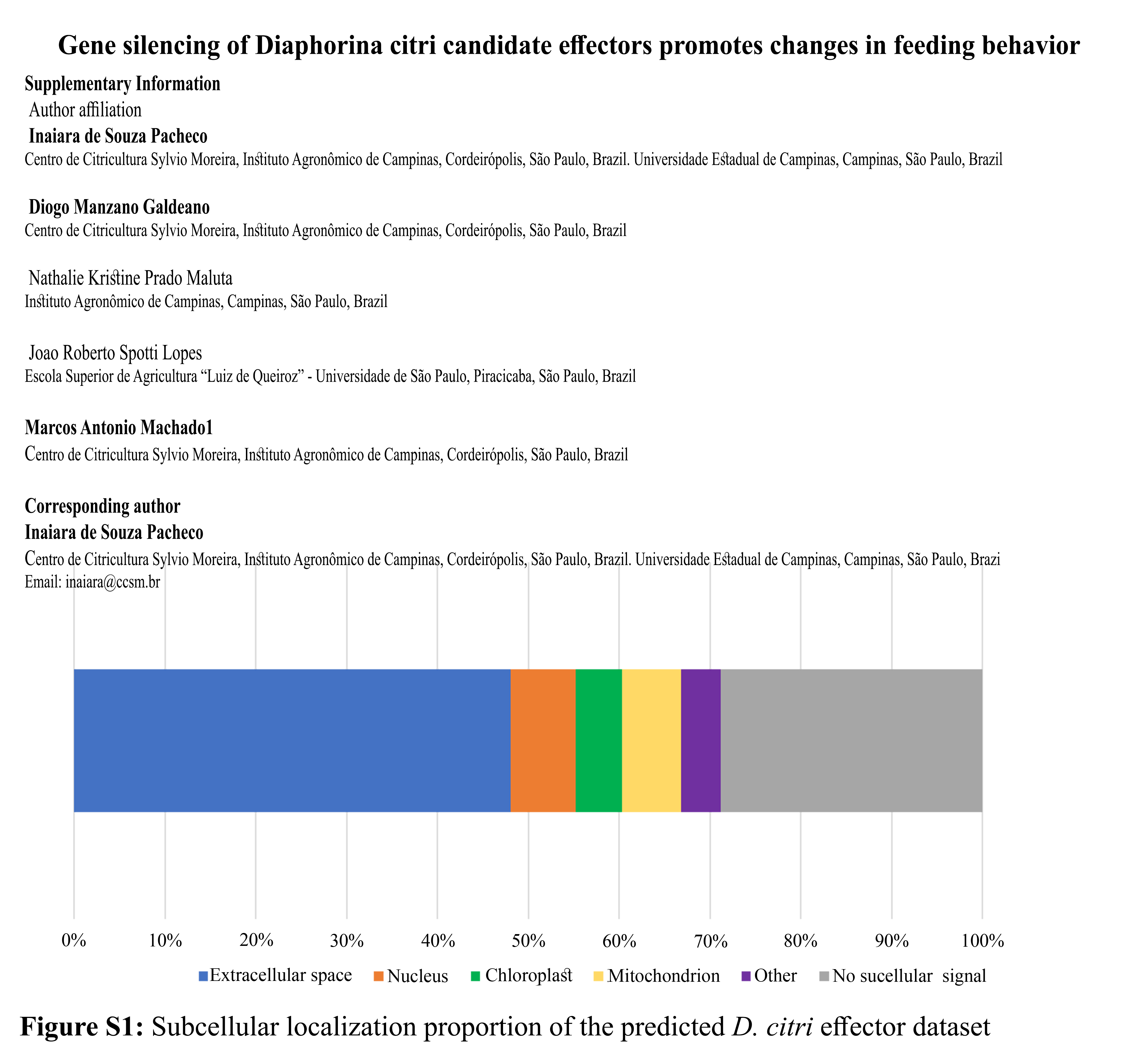

Supplement: Supplementary file 1 — supplementary information. [file 41598_2020_62856_MOESM1_ESM.tif]

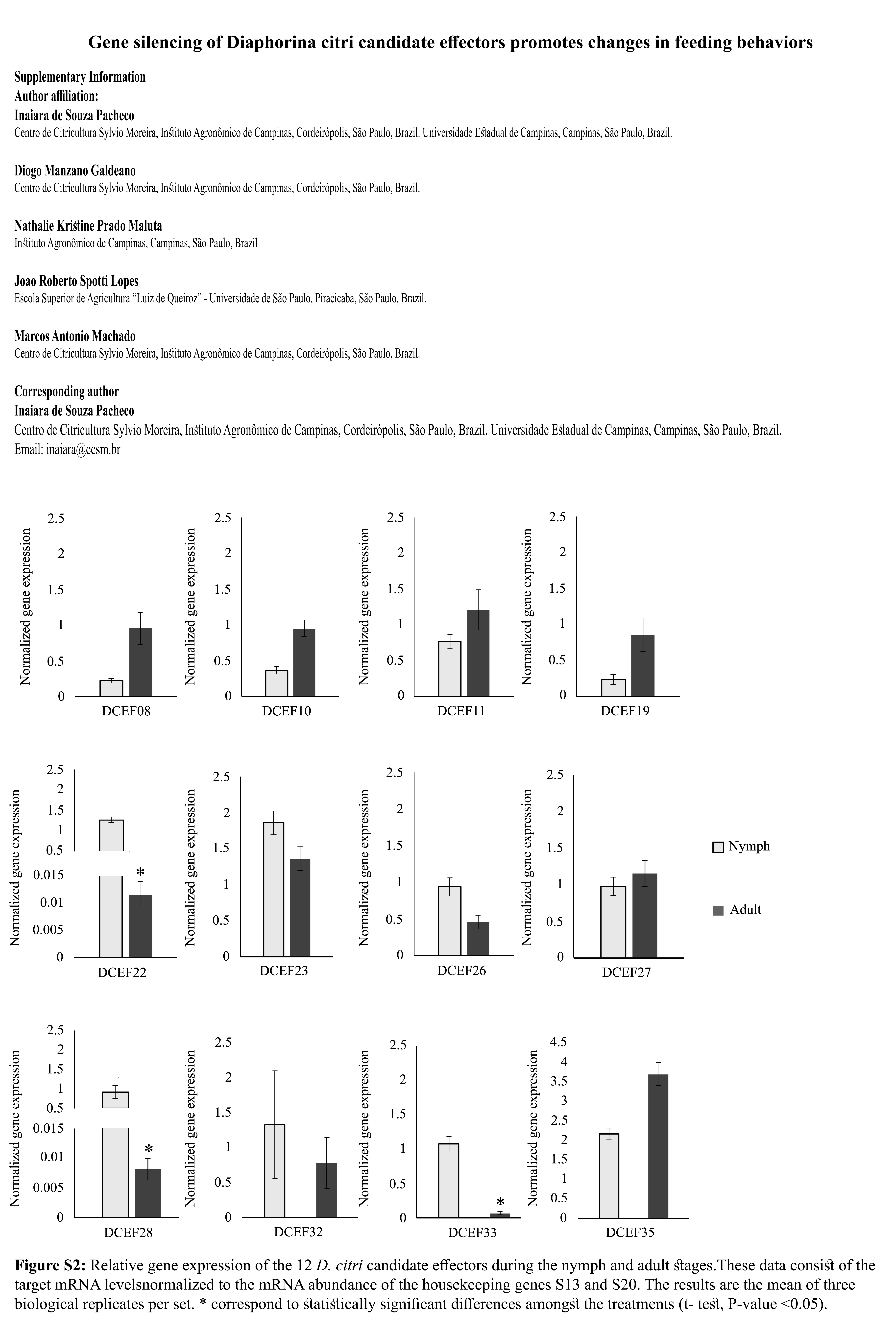

Supplement: Supplementary file 2 — supplementary information 2. [file 41598_2020_62856_MOESM2_ESM.tif]

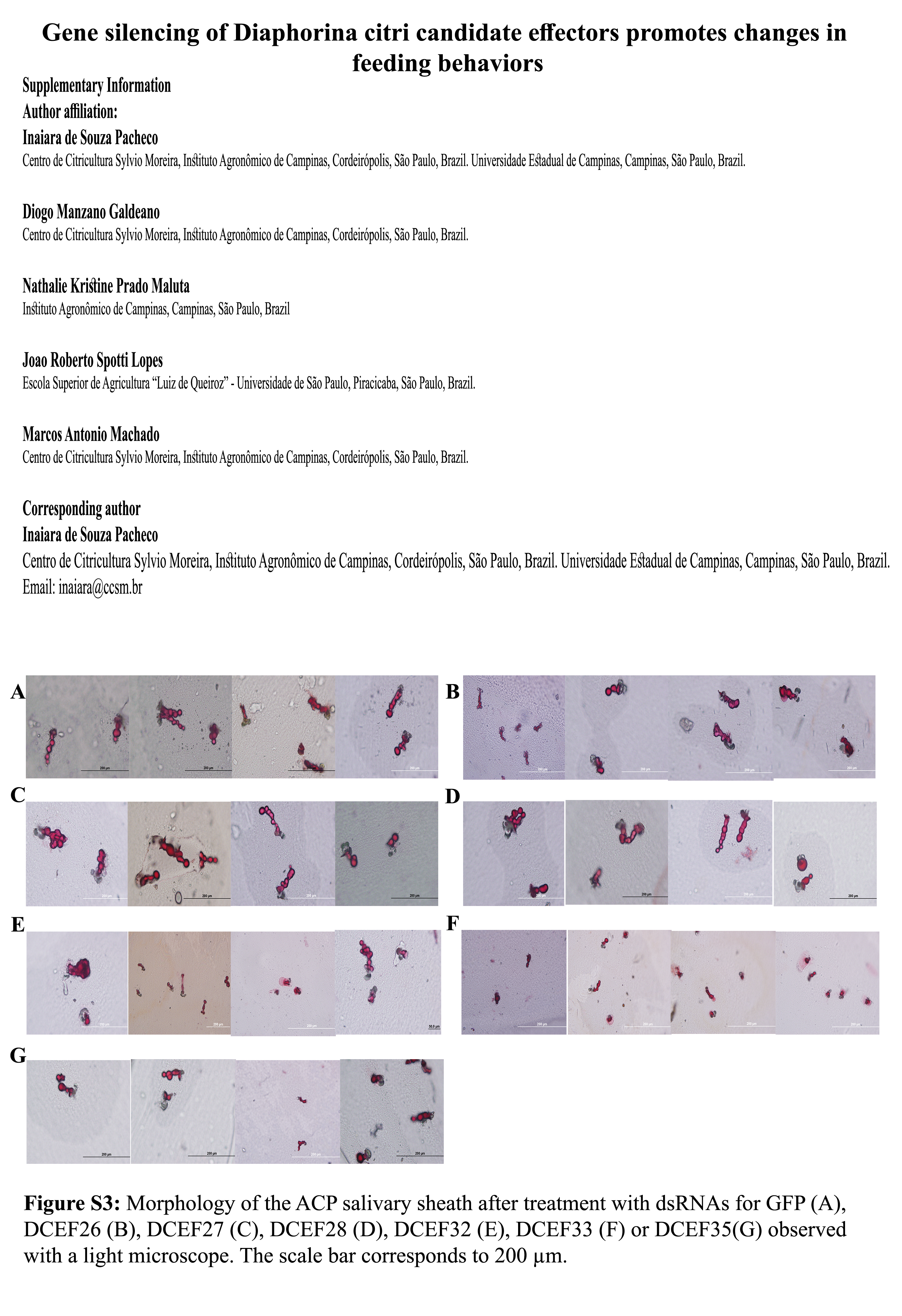

Supplement: Supplementary file 3 — supplementary information 3. [file 41598_2020_62856_MOESM3_ESM.tif]

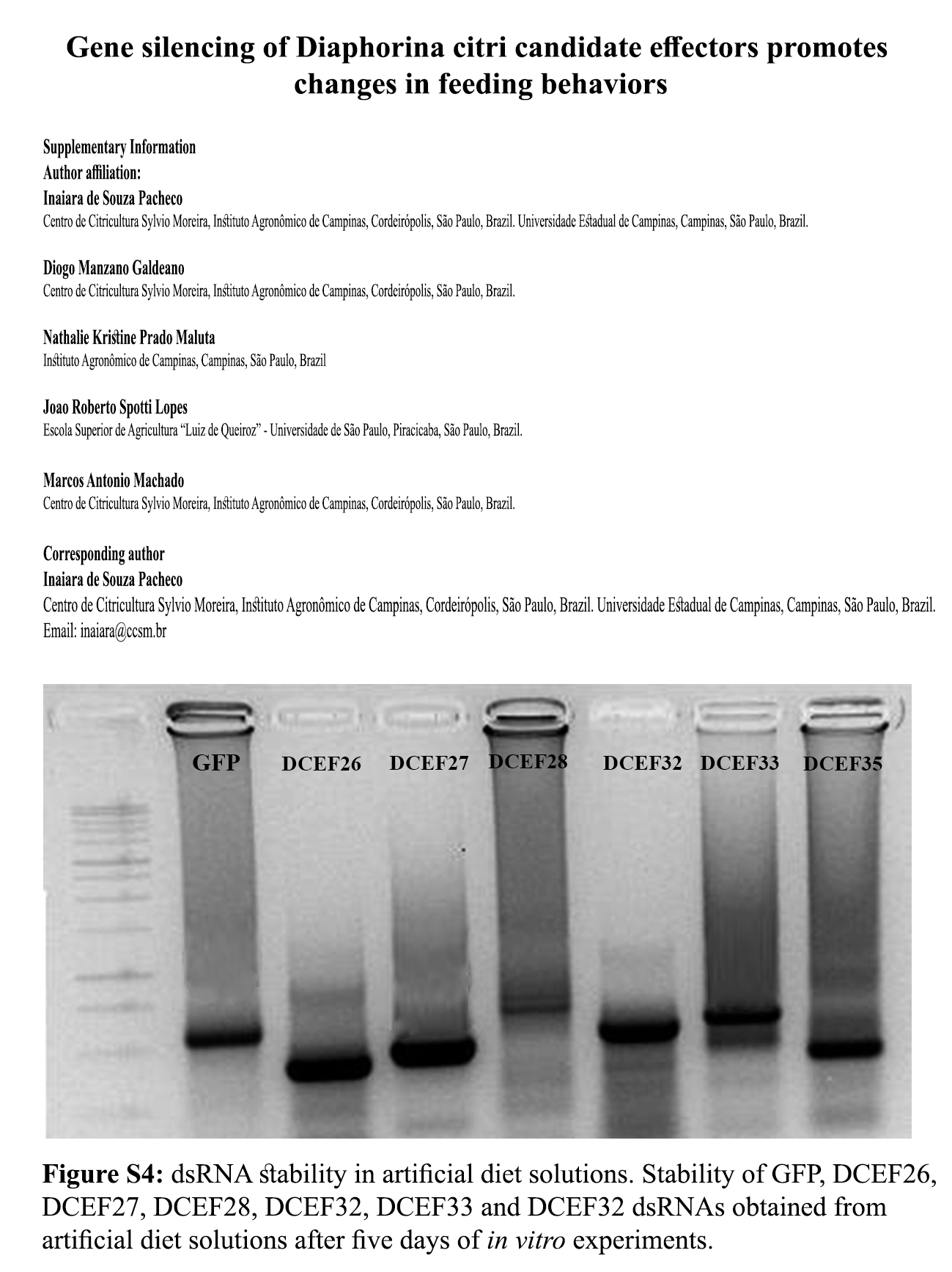

Supplement: Supplementary file 4 — supplementary information 4. [file 41598_2020_62856_MOESM4_ESM.tif]
