# Supplementary material for: Gene silencing of Diaphorina citri candidate effectors promotes changes in feeding behaviors
Source: Sci Rep. 2020 Apr 7;10:5992. doi: 10.1038/s41598-020-62856-5 (PMC7138822; doi:10.1038/s41598-020-62856-5)
Supplement: Supplementary file 5 — supplementary information 5. [file 41598_2020_62856_MOESM5_ESM.docx]

**Gene silencing of *Diaphorina citri* candidate effectors promotes changes in feeding behaviors**

**Supplementary Information**

**Author affiliation:**

**Inaiara de Souza Pacheco**

Centro de Citricultura Sylvio Moreira, Instituto Agronômico de Campinas, Cordeirópolis, São Paulo, Brazil. Universidade Estadual de Campinas, Campinas, São Paulo, Brazil.

**Diogo Manzano Galdeano**

Centro de Citricultura Sylvio Moreira, Instituto Agronômico de Campinas, Cordeirópolis, São Paulo, Brazil.

**Nathalie Kristine Prado Maluta**

Instituto Agronômico de Campinas, Campinas, São Paulo, Brazil

**Joao Roberto Spotti Lopes**

Escola Superior de Agricultura “Luiz de Queiroz” - Universidade de São Paulo, Piracicaba, São Paulo, Brazil.

**Marcos Antonio Machado**

Centro de Citricultura Sylvio Moreira, Instituto Agronômico de Campinas, Cordeirópolis, São Paulo, Brazil.

**Corresponding author**

**Inaiara de Souza Pacheco**

Centro de Citricultura Sylvio Moreira, Instituto Agronômico de Campinas, Cordeirópolis, São Paulo, Brazil. Universidade Estadual de Campinas, Campinas, São Paulo, Brazil.

Email: inaiara@ccsm.br

**Supplementary Table S1:** Predicted *D. citri* effectors from the genomic and transcriptomic databases using bioinformatic tools.

| **Effector** | **NCBI ID** | **Description** | **Domains** | **Subcellular localization** |
| --- | --- | --- | --- | --- |
| DCEF01 | XP_017299442.1 | uncharacterized protein LOC103508878 | Intrinsically disordered regions; non_cytoplasmic_domain; signal_peptide | Extracellular space |
| DCEF02 | XP_008487102.1 | uncharacterized protein LOC103523870 | Signal_peptide; non_cytoplasmic_domain; signalp-notm (signalp_euk) | Extracellular space |
| DCEF03 | XP_017304428.1 | uncharacterized protein LOC103522039 | Non_cytoplasmic_domain; signal_peptide; signalp-notm (signalp_euk) | - |
| DCEF04 | XP_017298003.1 | uncharacterized protein LOC103505648 | Intrinsically disordered regions; non_cytoplasmic_domain; signal_peptide; signalp-notm (signalp_euk) | Extracellular space |
| DCEF05 | XP_008482697.1 | uncharacterized protein LOC103519389 | Ipr013547; intrinsically disordered regions; signal_peptide; non_cytoplasmic_domain; signalp-tm (signalp_gram_positive); signalp-notm (signalp_euk); signalp-notm (signalp_gram_negative) | Extracellular space |
| DCEF06 | XP_008475221.1 | uncharacterized protein LOC103512248 | Ipr002557; ipr002557; pthr23301: sf42; pthr23301; non_cytoplasmic_domain; signal_peptide; ipr002557; signalp-notm (signalp_euk); ipr036508; | Extracellular space |
| DCEF07 | XP_008482131.1 | ---NA--- | Intrinsically disordered regions; signal_peptide; non_cytoplasmic_domain; signalp-notm (signalp_euk) | Extracellular space |
| DCEF08 | XP_008479196.1 | WD repeat-containing protein 92 | Ipr015943; pthr10971; pthr10971: sf2; ipr036322; | Extracellular space |
| DCEF09 | XP_017303346.1 | uncharacterized protein LOC103518581 | Intrinsically disordered regions; signal_peptide; non_cytoplasmic_domain; ps51257; signalp-notm (signalp_euk) | Extracellular space |
| DCEF10 | XP_008482426.1 | ---NA--- | Non_cytoplasmic_domain; signal_peptide; signalp-notm (signalp_euk) | - |
| DCEF11 | XP_026681229.1 | ---NA--- | Intrinsically disordered regions; non_cytoplasmic_domain; signal_peptide; signalp-notm (signalp_euk) | Mitochondrion/chloroplast |
| DCEF12 | XP_017303319.1 | uncharacterized protein LOC103518511 | Non_cytoplasmic_domain; signal_peptide; signalp-notm (signalp_euk); signalp-tm (signalp_gram_positive) | Chloroplast |
| DCEF13 | XP_008476916.1 | uncharacterized protein LOC103513847 | Non_cytoplasmic_domain; signalp-notm (signalp_euk) | - |
| DCEF14 | XP_008481488.1 | uncharacterized protein LOC103513847 | Non_cytoplasmic_domain; signalp-notm (signalp_euk) | Extracellular space |
| DCEF15 | XP_008481339.2 | uncharacterized protein LOC103518061 | Non_cytoplasmic_domain; signal_peptide; signalp-notm (signalp_euk) | Extracellular space |
| DCEF16 | DcWN_028088_1 | ---NA--- | Non_cytoplasmic_domain; signal_peptide; signalp-notm (signalp_euk) | Mitochondrion/chloroplast |
| DCEF17 | DcWN_030518_1 | ---NA--- | Signal_peptide; non_cytoplasmic_domain; signalp-notm (signalp_euk) | Extracellular space |
| DCEF18 | XP_008481275.1 | uncharacterized protein LOC103518002 | Non_cytoplasmic_domain; signal_peptide; signalp-notm (signalp_euk) | Extracellular space |
| DCEF19 | DcWN_006647_1 | ---NA--- | Non_cytoplasmic_domain; cytoplasmic domain; signalp-notm (signalp_euk) | Extracellular space |
| DCEF20 | XP_008481052.1 | uncharacterized protein LOC103517785 | Intrinsically disordered regions; signal_peptide; non_cytoplasmic_domain; signalp-notm (signalp_euk); signalp-tm (signalp_gram_positive) | Extracellular space |
| DCEF21 | XP_017303040.1 | uncharacterized protein LOC103517708 | Intrinsically disordered regions; non_cytoplasmic_domain; signal_peptide; signalp-tm (signalp_gram_positive); signalp-notm (signalp_euk) | Extracellular space |
| DCEF22 | DcWN_010436_1 | ---NA--- | Non_cytoplasmic_domain; signal_peptide; signalp-notm (signalp_euk) | - |
| DCEF23 | DcWN_013243_1 | ---NA--- | Non_cytoplasmic_domain; signal_peptide; signalp-notm (signalp_euk) | - |
| DCEF24 | XP_008480891.1 | uncharacterized protein LOC103517629 | Signal_peptide; non_cytoplasmic_domain; ps51257; signalp-notm (signalp_euk); signalp-notm (signalp_gram_negative); signalp-tm (signalp_gram_positive) | - |
| DCEF25 | XP_008479411.1 | trithorax group proteinosa-like | Intrinsically disordered regions; non cytoplasmic domain; signal_peptide; signalp-notm (signalp_gram_negative); signalp-notm (signalp_euk); signalp-tm (signalp_gram_positive) | Extracellular space |
| DCEF26 | DcWN_027964_1 | ---NA--- | Non_cytoplasmic_domain; transmembrane; cytoplasmic domain; signalp-notm (signalp_euk) | Mitochondrion |
| DCEF27 | DcWN_028357_1 | ---NA--- | Signalp-notm (signalp_euk) | Mitochondrion/chloroplast |
| DCEF28 | XP_026685929.1 | uncharacterized protein LOC113471181 | Intrinsically disordered regions; signalp-notm (signalp_euk) | - |
| DCEF29 | XP_017302380.1 | ---NA--- | Intrinsically disordered regions; | Extracellular space |
| DCEF30 | XP_017303664.1 | uncharacterized protein LOC103519362 | Ipr031942; intrinsically disordered regions; intrinsically disordered regions; non cytoplasmic domain; signal_peptide; signalp-notm (signalp_euk) | - |
| DCEF31 | XP_008473589.1 | KH domain-containing, RNA-binding, signal transduction-associated protein 2 | Intrinsically disordered regions; non_cytoplasmic_domain; signal_peptide; signalp-notm (signalp_euk) | Nucleus |
| DCEF32DCEF | XP_008477481.1 | receptor-binding cancer antigen expressed on SiSo cells | Ipr017025; intrinsically disordered regions; ipr017025; signal_peptide; non_cytoplasmic_domain; signalp-notm (signalp_euk) | Golgi apparatus |
| DCEF33 | XP_008475409.1 | secreted Lectin-like protein | Ipr016186; pthr21407: sf1; pthr21407: sf1; pthr21407; pthr21407; signal_peptide; non_cytoplasmic_domain; ipr001304; cd00037; signalp-notm (signalp_euk); signalp-tm (signalp_gram_positive); ipr016187; | Extracellular space |
| DCEF34 | XP_008472683.1 | gamma-hordein-3-like | Intrinsically disordered regions; signal_peptide; non_cytoplasmic_domain; signalp-tm (signalp_gram_positive); signalp-notm (signalp_euk) | Chloroplast |
| DCEF35 | XP_008468032.1 | uncharacterized protein LOC103505475 | - | Extracellular space |
| DCEF36 | XP_008478960.1 | uncharacterized protein LOC103515795 | Intrinsically disordered regions; non_cytoplasmic_domain; signal_peptide; signalp-tm (signalp_gram_positive); signalp-notm (signalp_euk) | - |
| DCEF37 | XP_008478834.1 | uncharacterized protein LOC103515675 | Non_cytoplasmic_domain; signal_peptide; signalp-notm (signalp_euk); signalp-tm (signalp_gram_positive) | Extracellular space |
| DCEF38 | XP_008478397.1 | uncharacterized protein LOC113469938 | Intrinsically disordered regions; ipr015510; pthr11022: sf44; non_cytoplasmic_domain; signal_peptide; signalp-tm (signalp_gram_positive); signalp-notm (signalp_euk) | Extracellular space |
| DCEF39 | XP_008478205.1 | uncharacterized protein LOC103515064 | Signal_peptide; non_cytoplasmic_domain; signalp-notm (signalp_euk); signalp-tm (signalp_gram_positive) | Extracellular space |
| DCEF40 | XP_008478019.1 | uncharacterized protein LOC103514880 | Signal_peptide; non_cytoplasmic_domain; signalp-notm (signalp_euk) | Extracellular space |
| DCEF41 | XP_008477753.1 | uncharacterized protein LOC103514633 | Intrinsically disordered regions; non_cytoplasmic_domain; signal_peptide; signalp-notm (signalp_euk) | Extracellular space |
| DCEF42 | XP_008477632.1 | uncharacterized protein LOC103514515 | Non_cytoplasmic_domain; signal_peptide; signalp-notm (signalp_euk); signalp-notm (signalp_gram_negative) | Extracellular space |
| DCEF43 | XP_008477419.1 | uncharacterized protein LOC103514327 | Intrinsically disordered regions; signal_peptide; non_cytoplasmic_domain; signalp-notm (signalp_euk); signalp-tm (signalp_gram_positive); signalp-notm (signalp_gram_negative) | - |
| DCEF44 | XP_017301576.1 | uncharacterized protein LOC103514111 | Intrinsically disordered regions; signal_peptide; non_cytoplasmic_domain; signalp-tm (signalp_gram_positive); signalp-notm (signalp_euk); signalp-notm (signalp_gram_negative) | - |
| DCEF45 | XP_008477078.1 | uncharacterized protein LOC103513992 | Intrinsically disordered regions; signal_peptide; non_cytoplasmic_domain; signalp-notm (signalp_euk) | - |
| DCEF46 | XP_008476866.1 | uncharacterized protein LOC103513797 | Non_cytoplasmic_domain; signal_peptide; signalp-notm (signalp_euk) | Extracellular space |
| DCEF47 | XP_008476773.1 | uncharacterized protein LOC103513701 | Signal_peptide; non_cytoplasmic_domain; signalp-notm (signalp_euk) | Nucleus |
| DCEF48 | XP_008476667.1 | uncharacterized protein LOC103513599 | Signal_peptide; non_cytoplasmic_domain; signalp-notm (signalp_euk) | - |
| DCEF49 | XP_008476659.1 | ---NA--- | Non_cytoplasmic_domain; signal_peptide; signalp-notm (signalp_euk) | Extracellular space |
| DCEF50 | XP_008476658.1 | uncharacterized protein LOC103513591 | Non_cytoplasmic_domain; signalp-notm (signalp_euk) | Extracellular space |
| DCEF51 | XP_008476584.1 | uncharacterized protein LOC103513525 | Intrinsically disordered regions; non_cytoplasmic_domain; signal_peptide; signalp-notm (signalp_euk) | - |
| DCEF52 | XP_017301283.1 | uncharacterized protein LOC103513379 | Non_cytoplasmic_domain; signal_peptide; signalp-notm (signalp_euk); signalp-notm (signalp_gram_negative); signalp-tm (signalp_gram_positive) | - |
| DCEF53 | XP_017301056.1 | uncharacterized protein LOC103512790 | Intrinsically disordered regions; non_cytoplasmic_domain; signal_peptide; signalp-notm (signalp_euk) | Extracellular space |
| DCEF54 | XP_008475522.1 | uncharacterized protein LOC103511975 | Signal_peptide; non_cytoplasmic_domain; signalp-notm (signalp_euk); signalp-tm (signalp_gram_positive) | Extracellular space |
| DCEF55 | XP_017300636.1 | uncharacterized protein PFB0145c-like | Intrinsically disordered regions; non_cytoplasmic_domain; signal_peptide; signalp-notm (signalp_euk); signalp-notm (signalp_gram_negative) | - |
| DCEF56 | XP_008474372.2 | uncharacterized protein LOC103511425 | Intrinsically disordered regions; signal_peptide; non_cytoplasmic_domain; signalp-notm (signalp_euk); signalp-tm (signalp_gram_positive) | - |
| DCEF57 | XP_008474088.1 | uncharacterized protein LOC103511151 | Intrinsically disordered regions; signal_peptide; non_cytoplasmic_domain; signalp-tm (signalp_gram_positive); signalp-notm (signalp_euk) | - |
| DCEF58 | XP_017300104.1 | uncharacterized protein LOC103510523 | Signal_peptide; non_cytoplasmic_domain; signalp-notm (signalp_euk) | - |
| DCEF59 | XP_017300048.1 | uncharacterized protein LOC113465279 | Non_cytoplasmic_domain; signal_peptide; signalp-tm (signalp_gram_positive); signalp-notm (signalp_euk); signalp-notm (signalp_gram_negative) | - |
| DCEF60 | XP_017299977.1 | uncharacterized protein LOC103510221 | Intrinsically disordered regions; non_cytoplasmic_domain; signal_peptide; signalp-notm (signalp_euk) | - |
| DCEF61 | XP_008477626.1 | basic salivary proline-rich protein 3-like | - | Extracellular space |
| DCEF62 | XP_017299931.1 | uncharacterized protein LOC103510101 | Non_cytoplasmic_domain; signal_peptide; signalp-notm (signalp_euk) | Extracellular space |
| DCEF63 | XP_008472484.2 | uncharacterized protein LOC103509637 | Non_cytoplasmic_domain; signal_peptide; signalp-notm (signalp_gram_negative); signalp-notm (signalp_euk) | Extracellular space |
| DCEF64 | XP_017299728.1 | uncharacterized protein LOC103509593 | Intrinsically disordered regions; signal_peptide; non_cytoplasmic_domain; signalp-tm (signalp_gram_positive); signalp-notm (signalp_euk) | Extracellular space |
| DCEF65 | XP_008472279.1 | putative odorant-binding protein | Intrinsically disordered regions; non_cytoplasmic_domain; signal_peptide; signalp-notm (signalp_euk); signalp-tm (signalp_gram_positive) | Extracellular space |
| DCEF66 | XP_017299608.1 | uncharacterized protein LOC103509257 | Signal_peptide; non_cytoplasmic_domain; signalp-notm (signalp_euk) | Extracellular space |
| DCEF67 | XP_017299566.1 | endocuticle structural glycoprotein SgAbd-8-like | Ipr000618; signal_peptide; non_cytoplasmic_domain; ipr000618; signalp-notm (signalp_euk) | Extracellular space |
| DCEF68 | XP_008471868.1 | uncharacterized protein LOC103509057 | Signal_peptide; non_cytoplasmic_domain; signalp-notm (signalp_euk) | Extracellular space |
| DCEF69 | XP_017298352.1 | adhesive plaque matrix protein-like | Intrinsically disordered regions; signal_peptide; non_cytoplasmic_domain; signalp-notm (signalp_euk) | Nucleus |
| DCEF70 | XP_017298164.1 | uncharacterized protein LOC103506019 | Intrinsically disordered regions; signal_peptide; non_cytoplasmic_domain; signalp-notm (signalp_euk) | - |
| DCEF71 | XP_008468468.1 | uncharacterized protein LOC103505874 | Intrinsically disordered regions; signal_peptide; non_cytoplasmic_domain; ps51257; signalp-notm (signalp_euk) | - |
| DCEF72 | XP_017305383.1 | uncharacterized protein LOC103504916 | Intrinsically disordered regions; pthr11861; signal_peptide; non_cytoplasmic_domain; signalp-notm (signalp_gram_negative); signalp-tm (signalp_gram_positive); signalp-notm (signalp_euk) | Extracellular space |
| DCEF73 | XP_008487661.1 | uncharacterized protein LOC103524424 | Intrinsically disordered regions; signal_peptide; non_cytoplasmic_domain; signalp-notm (signalp_euk); signalp-notm (signalp_gram_negative) | Nucleus |
| DCEF74 | XP_017305023.1 | uncharacterized protein LOC103524236 | Intrinsically disordered regions; signal_peptide; non_cytoplasmic_domain; signalp-notm (signalp_euk) | Nucleus |
| DCEF75 | XP_008476900.1 | uncharacterized protein LOC103513825 isoform | Non_cytoplasmic_domain; signal_peptide; signalp-tm (signalp_gram_positive); signalp-notm (signalp_euk); signalp-notm (signalp_gram_negative) | Extracellular space |
| DCEF76 | XP_008480626.1 | uncharacterized protein LOC103517374 | Ipr011042; non_cytoplasmic_domain; signal_peptide; signalp-notm (signalp_euk); ssf63825; | Extracellular space |
| DCEF77 | XP_008472531.1 | uncharacterized protein LOC103509681 | Ipr002557; pthr22933: sf12; pthr22933; pthr22933: sf12; pthr22933; signal_peptide; non_cytoplasmic_domain; ipr002557; signalp-notm (signalp_euk); signalp-notm (signalp_gram_negative); signalp-tm (signalp_gram_positive); ipr036508; | Extracellular space |
| DCEF78 | XP_008481816.1 | uncharacterized protein LOC103518520 | Ipr000884; ipr036383; ipr000884; pthr11311: sf15; pthr11311; signal_peptide; non_cytoplasmic_domain; ipr000884; signalp-notm (signalp_euk); ipr036383; | Extracellular space |
| DCEF79 | diaci_adult_77770000001671_1 | ---NA--- | Intrinsically disordered regions; non_cytoplasmic_domain; cytoplasmic domain | - |
| DCEF80 | diaci_adult_77770000002706_1 | ---NA--- | - | Nucleus |
| DCEF81 | XP_008482489.1 | uncharacterized protein LOC103519182 | Ipr006170; ipr006170; ipr036728; pthr11857; signal_peptide; non_cytoplasmic_domain; signalp-tm (signalp_gram_positive); signalp-notm (signalp_gram_negative); signalp-notm (signalp_euk); ipr036728; | Extracellular space |
| DCEF82 | XP_008474286.2 | uncharacterized protein LOC103511341 | Intrinsically disordered regions; | Nucleus |
| DCEF83 | XP_017300350.1 | endoplasmic homolog | Ipr020575; ipr003594; ipr001404; ipr001404; ipr036890 ipr003594; ipr037196; intrinsically disordered regions; pthr11528: sf73; ipr001404; non_cytoplasmic_domain; signal_peptide; ipr001404; ipr003594; signalp-notm (signalp_euk); ipr020568; ipr037196; ipr036890; | Endoplasmic reticulum |
| DCEF84 | diaci_adult_77770000013294_1 | ---NA--- | Intrinsically disordered regions; | Mitochondrion |
| DCEF85 | XP_026676319.1 | uncharacterized protein LOC113465746 | - | Mitochondrion |
| DCEF86 | diaci_adult_77770000019294_1 | ---NA--- | Intrinsic disorder region | Cytoplasm |
| DCEF87 | diaci_adult_77770000027404_1 | ---NA--- | Intrinsically disordered regions; | Nucleus |
| DCEF88 | XP_008483070.1 | otolith matrix protein OMM-64-like | Signalp-notm (signalp_euk); signalp-tm (signalp_gram_positive) | Nucleus |
| DCEF89 | XP_008471618.1 | putative defense protein Hdd11 | Ipr002861; pthr23130; pthr23130: sf122; signal_peptide; non_cytoplasmic_domain; ipr002861; ipr002861; signalp-notm (signalp_gram_negative); signalp-notm (signalp_euk); signalp-tm (signalp_gram_positive) | Extracellular space |
| DCEF90 | XP_017302787.2 | uncharacterized protein LOC103517027 | Intrinsically disordered regions; | - |
| DCEF91 | XP_008477226.1 | alpha-L-fructosidase | Ipr016286; ipr000933; ipr031919; ipr000933; ipr016286; ipr000933; pthr10030: sf40; signal_peptide; non_cytoplasmic_domain; signalp-notm (signalp_euk); signalp-tm (signalp_gram_positive); ipr017853; | - |
| DCEF92 | XP_008483858.1 | uncharacterized protein LOC103520538 | Ipr001604; ipr020821; ipr001604; ipr040255; pthr13966: sf11; non_cytoplasmic_domain; signal_peptide; signalp-notm (signalp_euk); ssf54060; | Extracellular space |
| DCEF93 | diaci_nymph_66660000001344_1 | ---NA--- | Signalp-notm (signalp_euk); signalp-tm (signalp_gram_positive) | Mitochondrion |
| DCEF94 | XP_017304622.1 | cell-derived factor 2 | Ipr016093; ipr016093; pthr10050: sf46; ipr027005; ipr016093; ipr016093; signalp-notm (signalp_euk); ipr036300; | Extracellular space |
| DCEF95 | XP_008468734.1 | cuticle protein 7-like | Pr01217; ipr000618; intrinsically disordered regions; pthr12236; pthr12236: sf46; non_cytoplasmic_domain; signal_peptide; ipr000618; signalp-notm (signalp_gram_negative); signalp-tm (signalp_gram_positive); signalp-notm (signalp_euk) | Extracellular space |
| DCEF96 | XP_026687867.1 | venom carboxylesterase-6-like | Ipr002018; ipr029058; pthr11559: sf347; pthr11559: sf347; pthr11559; pthr11559; pthr11559; pthr11559: sf347; non_cytoplasmic_domain; signal_peptide; signalp-notm (signalp_euk); ipr029058; | - |
| DCEF97 | XP_008480789.2 | LOW QUALITY PROTEIN: protein takeout-like | Ipr010562; ipr010562; ipr038606; intrinsically disordered regions; pthr11008; non_cytoplasmic_domain; signal_peptide; signalp-notm (signalp_euk); signalp-notm (signalp_gram_negative) | Extracellular space |
| DCEF98 | XP_008482920.1 | beta-galactosidase | Ipr001944; ipr008979; ipr026283; ipr008979; ipr031330; pthr23421: sf83; ipr001944; non_cytoplasmic_domain; signal_peptide; signalp-notm (signalp_euk); signalp-tm (signalp_gram_positive); ipr008979; ipr017853; | Lysosome |
| DCEF99 | XP_026680053.1 | protein takeout-like | Ipr010562; ipr010562; ipr038606; pthr11008; non_cytoplasmic_domain; signal_peptide; signalp-notm (signalp_euk) | Extracellular space |
| DCEF100 | XP_026675931.1 | uncharacterized protein LOC103524877 | Ipr032062; non_cytoplasmic_domain; signal_peptide; signalp-notm (signalp_euk) | Extracellular space |
| DCEF101 | XP_026682292.1 | protein disulfide-isomerase-like | Pr00421; pf13848; ipr013766; ipr005788; pthr18929; pthr18929: sf101; signal_peptide; non_cytoplasmic_domain; ipr013766; cd02961; cd02981; signalp-notm (signalp_euk); signalp-tm (signalp_gram_positive); signalp-notm (signalp_gram_negative); ipr036249; ipr036249; ipr036249; | Endoplasmic reticulum |
| DCEF102 | XP_026684010 | lysosomal acid phosphatase-like | Ipr029033; ipr000560; pthr11567; pthr11567: sf170; signal_peptide; non_cytoplasmic_domain; ipr000560; signalp-notm (signalp_gram_negative); signalp-tm (signalp_gram_positive); signalp-notm (signalp_euk); ipr029033; | - |
| DCEF103 | XP_026684575.1 | uncharacterized protein LOC103516340 | Non_cytoplasmic_domain; signalp-notm (signalp_gram_negative); signalp-tm (signalp_gram_positive); signalp-notm (signalp_euk); ssf57302; | Extracellular space |
| DCEF104 | XP_026686493.1 | uncharacterized protein LOC103519085 | Non_cytoplasmic_domain; ps51257; signalp-notm (signalp_euk) | - |
| DCEF105 | XP_026687640.1 | ER degradation-enhancing alpha-mannosidase-like protein 2 | Ipr001382; ipr001382; ipr012341; intrinsically disordered regions; pthr11742: sf41; pthr11742; signal_peptide; non_cytoplasmic_domain; signalp-notm (signalp_euk); ipr036026; | Endoplasmic reticulum |
| DCEF106 | XP_026675910.1 | uncharacterized protein LOC103524856 | Ipr032062; ipr029058; ipr002018; intrinsically disordered regions; pthr43918; pthr43918: sf3; non_cytoplasmic_domain; signal_peptide; signalp-notm (signalp_euk); ipr029058; | Extracellular space |
| DCEF107 | NP_001316109.1 | cathepsin B-like precursor | Ipr000668; ipr000668; ipr012599; ipr000668; intrinsically disordered regions; pthr12411: sf540; ipr013128; signal_peptide; non_cytoplasmic_domain; cd02620; signalp-notm (signalp_euk); ipr038765; | - |
| DCEF108 | XP_008475754.2 | uncharacterized protein LOC103512753 | Signal_peptide; non_cytoplasmic_domain; signalp-tm (signalp_gram_positive); signalp-notm (signalp_euk) | Mitochondrion/chloroplast |
| DCEF109 | XP_026685776.1 | carbonic anhydrase 4-like | Ipr001148; ipr036398; ipr001148; ipr023561; pthr18952: sf183; non_cytoplasmic_domain; signal_peptide; ipr001148; cd00326; signalp-notm (signalp_euk); ipr036398; | - |
| DCEF110 | XP_017299489.1 | regulator of microtubule dynamics protein 2-like | Ipr011990; ipr011990; pthr16056; pthr16056: sf16; signal_peptide; non_cytoplasmic_domain; signalp-notm (signalp_euk); ipr011990; | - |
| DCEF111 | XP_008467481.1 | uncharacterized protein LOC103504956 | Signal_peptide; non_cytoplasmic_domain; ps51257; signalp-notm (signalp_gram_negative); signalp-notm (signalp_euk); signalp-tm (signalp_gram_positive) | Extracellular space |
| DCEF112 | XP_008468057.1 | uncharacterized protein LOC103505498 | Non_cytoplasmic_domain; signal_peptide; signalp-notm (signalp_euk) | Extracellular space |
| DCEF113 | XP_008469045.1 | uncharacterized protein LOC103506434 | Intrinsically disordered regions; signal_peptide; non_cytoplasmic_domain; signalp-notm (signalp_gram_negative); signalp-notm (signalp_euk) | Extracellular space |
| DCEF114 | XP_008469405.1 | uncharacterized protein LOC103506779 | Intrinsically disordered regions; signal_peptide; non_cytoplasmic_domain; signalp-notm (signalp_euk) | - |
| DCEF115 | XP_008469597.1 | uncharacterized protein LOC103506945 | Non_cytoplasmic_domain; signal_peptide; signalp-tm (signalp_euk) | Extracellular space |
| DCEF116 | XP_008470863.1 | ---NA--- | Non_cytoplasmic_domain; signal_peptide; signalp-notm (signalp_euk) | Extracellular space |
| DCEF117 | XP_008470924.1 | ---NA--- | Intrinsically disordered regions; non_cytoplasmic_domain; signal_peptide; signalp-notm (signalp_euk); signalp-notm (signalp_gram_negative); signalp-tm (signalp_gram_positive) | Nucleus |
| DCEF118 | XP_008471218.1 | uncharacterized protein LOC103508449 | Signal_peptide; non_cytoplasmic_domain; signalp-notm (signalp_euk); signalp-notm (signalp_gram_negative); signalp-tm (signalp_gram_positive); | Extracellular space |
| DCEF119 | XP_008471501.1 | uncharacterized protein LOC103508707 | Signal_peptide; non_cytoplasmic_domain; signalp-notm (signalp_euk) | Extracellular space |
| DCEF120 | XP_008471535.1 | uncharacterized protein LOC103508742 | Signal_peptide; non_cytoplasmic_domain; signalp-notm (signalp_gram_negative); signalp-notm (signalp_euk); signalp-tm (signalp_gram_positive) | Extracellular space |
| DCEF121 | XP_008472346.1 | uncharacterized protein LOC103509500 | Intrinsically disordered regions intrinsically disordered regions; non cytoplasmic domain; signal_peptide; signalp-tm (signalp_gram_positive); signalp-notm (signalp_euk) | - |
| DCEF122 | XP_008473435.1 | uncharacterized protein LOC109033602 | Pthr21398; pthr21398: sf19; pthr21398: sf19; signal_peptide; non_cytoplasmic_domain; signalp-tm (signalp_gram_positive); signalp-notm (signalp_euk) | Mitochondrion/chloroplast |
| DCEF123 | XP_008474869.1 | uncharacterized protein LOC103511903 | Signal_peptide; non_cytoplasmic_domain; signalp-notm (signalp_euk) | Extracellular space |
| DCEF124 | XP_008476188.1 | uncharacterized protein LOC103513157 | Intrinsically disordered regions; non_cytoplasmic_domain; signal_peptide; signalp-notm (signalp_euk) | - |
| DCEF125 | XP_008476361.1 | uncharacterized protein LOC103513319 | Intrinsically disordered regions; signal_peptide; non_cytoplasmic_domain; signalp-tm (signalp_gram_positive); signalp-notm (signalp_gram_negative); signalp-notm (signalp_euk) | - |
| DCEF126 | XP_008476423.1 | putative uncharacterized protein DDB_G0277057 | Intrinsically disordered regions; signal_peptide; non_cytoplasmic_domain; signalp-notm (signalp_euk) | Extracellular space |
| DCEF127 | XP_008481368.1 | neuropeptide NTL | Signal_peptide; non_cytoplasmic_domain; signalp-notm (signalp_euk) | Extracellular space |
| DCEF128 | XP_008483140.1 | uncharacterized protein LOC103519830 | Non_cytoplasmic_domain; signal_peptide; signalp-tm (signalp_gram_positive); signalp-notm (signalp_euk); | - |
| DCEF129 | XP_008484184.1 | uncharacterized protein LOC103520864 | Signal_peptide; non_cytoplasmic_domain; signalp-notm (signalp_euk) | Extracellular space |
| DCEF130 | XP_008486085.1 | Vg6-like protein | Signal_peptide; non_cytoplasmic_domain; signalp-notm (signalp_gram_negative); signalp-notm (signalp_euk) | Extracellular space |
| DCEF131 | XP_017301539.2 | adhesive plaque matrix protein-like | Non_cytoplasmic_domain; signal_peptide; signalp-tm (signalp_gram_positive); signalp-notm (signalp_gram_negative); signalp-notm (signalp_euk) | - |
