# Supplementary material for: Gene silencing of Diaphorina citri candidate effectors promotes changes in feeding behaviors
Source: Sci Rep. 2020 Apr 7;10:5992. doi: 10.1038/s41598-020-62856-5 (PMC7138822; doi:10.1038/s41598-020-62856-5)
Supplement: Supplementary file 6 — supplementary information 6. [file 41598_2020_62856_MOESM6_ESM.docx]

**Gene silencing of *Diaphorina citri* candidate effectors promotes changes in feeding behaviors**

**Supplementary Information**

**Author affiliation:**

**Inaiara de Souza Pacheco**

Centro de Citricultura Sylvio Moreira, Instituto Agronômico de Campinas, Cordeirópolis, São Paulo, Brazil. Universidade Estadual de Campinas, Campinas, São Paulo, Brazil.

**Diogo Manzano Galdeano**

Centro de Citricultura Sylvio Moreira, Instituto Agronômico de Campinas, Cordeirópolis, São Paulo, Brazil.

**Nathalie Kristine Prado Maluta**

Instituto Agronômico de Campinas, Campinas, São Paulo, Brazil

**Joao Roberto Spotti Lopes**

Escola Superior de Agricultura “Luiz de Queiroz” - Universidade de São Paulo, Piracicaba, São Paulo, Brazil.

**Marcos Antonio Machado**

Centro de Citricultura Sylvio Moreira, Instituto Agronômico de Campinas, Cordeirópolis, São Paulo, Brazil.

**Corresponding author**

**Inaiara de Souza Pacheco**

Centro de Citricultura Sylvio Moreira, Instituto Agronômico de Campinas, Cordeirópolis, São Paulo, Brazil. Universidade Estadual de Campinas, Campinas, São Paulo, Brazil.

Email: inaiara@ccsm.br

**Supplementary Table S2:** *D. citri* effectors that presented similarity with arthropod salivary gland proteins.

| **Effector** | **Description** | **e-Value** | **sim mean%** |
| --- | --- | --- | --- |
| DCEF01 | MpSG_ag2_G12 Myzus persicae, tobacco lineage, aphid salivary gland library Myzus persicae cDNA clone MpSG_ag2_G12, mRNA sequence | 7,12978E-14 | 54.16 |
| DCEF08 | CMRC-FF-IQ1-adf-m-03-0-CMRC.r1 Ceratitis capitata adult testes and male accessory gland cDNA library Ceratitis capitata cDNA 5', mRNA sequence | 4,5147E-74 | 72.77 |
| DCEF31 | FM983784 T. brucei infected salivary gland Glossina morsitans cDNA clone GMsg164a11.q1k, mRNA sequence | 5,2774E-51 | 69.18 |
| DCEF32 | MDEST1800 Hessian fly salivary gland cDNA Library Mayetiola destructor cDNA, mRNA sequence | 1,95316E-12 | 55.21 |
| DCEF33 | C03_RPL_P19 Fifth instar salivary gland cDNA library RPSG-1 Rhodnius prolixus cDNA, mRNA sequence | 3,2419E-123 | 62.8 |
| DCEF72 | E12_RPL_P21 Fifth instar salivary gland cDNA library RPSG-1 Rhodnius prolixus cDNA, mRNA sequence | 2,09859E-18 | 58.62 |
| DCEF75 | MDEST596 Hessian fly salivary gland cDNA Library Mayetiola destructor cDNA, mRNA sequence | 3,80814E-20 | 75.32 |
| DCEF77 | MDEST1183 Hessian fly salivary gland cDNA Library Mayetiola destructor cDNA, mRNA sequence | 2,54549E-53 | 53.65 |
| DCEF78 | FM959793 T. brucei infected salivary gland Glossina morsitans cDNA clone GMsg49h05.p1k, mRNA sequence | 1,34656E-25 | 57.91 |
| DCEF81 | 24d03 Pea Aphid Salivary Gland Acyrthosiphon pisum cDNA, mRNA sequence | 1,51312E-25 | 57.56 |
| DCEF83 | CTAR-P3-C08 Adult female salivary gland cDNA library Ctarsg1 Culex tarsalis cDNA, mRNA sequence | 1,5477E-94 | 76.03 |
| DCEF89 | 14h08 Pea Aphid Salivary Gland Acyrthosiphon pisum cDNA, mRNA sequence | 1,52827E-33 | 55.44 |
| DCEF91 | TMSGM_P6-970987_091_C11 Salivary gland cDNA library T_mat_sg1 Triatoma matogrossensis cDNA, mRNA sequence | 4,019E-109 | 80.,15 |
| DCEF92 | OlaSG_05-E09 Orius laevigatus salivary gland-specific EST library Orius laevigatus cDNA, mRNA sequence | 2,62456E-56 | 55.48 |
| DCEF94 | SCF-PPTNCSG_P8_L6_A06 Adult female Phlebotomus papatasi salivary gland library PPTNC Phlebotomus papatasi cDNA, mRNA sequence | 1,9442E-70 | 70.08 |
| DCEF95 | B06_RPM_P23 Fifth instar salivary gland cDNA library RPSG-1 Rhodnius prolixus cDNA, mRNA sequence | 4,68313E-14 | 77.46 |
| DCEF96 | DC551362 phe- Bombyx mori cDNA clone E_FL_phe-_24K18_F_0 5', mRNA sequence | 2,02137E-51 | 52.42 |
| DCEF97 | HA-GLN-49-libF_P24 Har-GLN norm Helicoverpa armigera cDNA, mRNA sequence | 2,12639E-20 | 52.13 |
| DCEF98 | ESG011b.E7_B09.5prime ESG01 Drosophila melanogaster cDNA 5' CT10342, mRNA sequence | 5,94096E-68 | 62.98 |
| DCEF99 | HV-PGN-384-05_P19 Hvir PG norm Heliothis virescens cDNA, mRNA sequence | 5,75586E-32 | 51.96 |
| DCEF100 | HA_MX1_62a02_SP6 Lobster Multiple Tissues, Normalized Homarus americanus cDNA clone HA_MX1_62a02 5' ref | 6,68992E-56 | 56.74 |
| DCEF101 | TMSGM_P7-971077_043_C05 Salivary gland cDNA library T_mat_sg1 Triatoma matogrossensis cDNA, mRNA sequence | 8,8923E-106 | 70.85 |
| DCEF102 | MDEST465 Hessian fly salivary gland cDNA Library Mayetiola destructor cDNA, mRNA sequence | 3,58652E-30 | 52.06 |
| DCEF103 | HA-GLN-384-07-libF_I09 Har-GLN norm Helicoverpa armigera cDNA, mRNA sequence | 1,01124E-47 | 70.18 |
| DCEF105 | HA-GLN-41-libF_H04 Har-GLN norm Helicoverpa armigera cDNA, mRNA sequence | 4,7401E-112 | 65.48 |
| DCEF107 | FM960354 T. brucei infected salivary gland Glossina morsitans cDNA clone GMsg05f10.p1k, mRNA sequence | 6,57017E-67 | 57.43 |
| DCEF109 | C10_C10sg1n19_pDNRf_515506 Myzus persicae, tobacco lineage, aphid salivary gland library Myzus persicae cDNA clone C10_C10sg1n19_pDNRf_515506, mRNA sequence | 2,63724E-21 | 48.96 |
| DCEF110 | SCF-PPTNCSG_P4_M2_A10 Adult female Phlebotomus papatasi salivary gland library PPTNC Phlebotomus papatasi cDNA, mRNA sequence | 3,08162E-13 | 51.59 |
| DCEF128 | DC548881 phe- Bombyx mori cDNA clone E_FL_phe-_15K13_F_0 5', mRNA sequence | 1,2236E-08 | 62.36 |
