# Supplementary material for: Gene silencing of Diaphorina citri candidate effectors promotes changes in feeding behaviors
Source: Sci Rep. 2020 Apr 7;10:5992. doi: 10.1038/s41598-020-62856-5 (PMC7138822; doi:10.1038/s41598-020-62856-5)
Supplement: Supplementary file 7 — supplementary information 7. [file 41598_2020_62856_MOESM7_ESM.docx]

**Gene silencing of *Diaphorina citri* candidate effectors promotes changes in feeding behaviors**

**Supplementary Information**

**Author affiliation:**

**Inaiara de Souza Pacheco**

Centro de Citricultura Sylvio Moreira, Instituto Agronômico de Campinas, Cordeirópolis, São Paulo, Brazil. Universidade Estadual de Campinas, Campinas, São Paulo, Brazil.

**Diogo Manzano Galdeano**

Centro de Citricultura Sylvio Moreira, Instituto Agronômico de Campinas, Cordeirópolis, São Paulo, Brazil.

**Nathalie Kristine Prado Maluta**

Instituto Agronômico de Campinas, Campinas, São Paulo, Brazil

**Joao Roberto Spotti Lopes**

Escola Superior de Agricultura “Luiz de Queiroz” - Universidade de São Paulo, Piracicaba, São Paulo, Brazil.

**Marcos Antonio Machado**

Centro de Citricultura Sylvio Moreira, Instituto Agronômico de Campinas, Cordeirópolis, São Paulo, Brazil.

**Corresponding author**

**Inaiara de Souza Pacheco**

Centro de Citricultura Sylvio Moreira, Instituto Agronômico de Campinas, Cordeirópolis, São Paulo, Brazil. Universidade Estadual de Campinas, Campinas, São Paulo, Brazil.

Email: inaiara@ccsm.br

**Supplementary Table S3:** Primer sequences for gene expression analysis for the twelve *D. citri* candidate effectors

| **Effector** | **ID** | **Primer sequence** |
| --- | --- | --- |
| DCEF08 | XP_008479196.1 | Forward-GCTGATTGAGAGGCTGAGG |
|  |  | Reverse-CACAACCCATCAATAGTCTGGA |
| DCEF10 | XP_008482426.1 | Forward-TGAGACCATACCCACCAGAAG |
|  |  | Reverse-TGCTGGAGAAATTATCCTCCTC |
| DCEF11 | XP_026681229.1 | Forward-GGTTCCGGGAATTATTCGTA |
|  |  | Reverse-GGGTCGTTAGTGGACTCGTG |
| DCEF19 | DcWN_006647 | Forward- GCTTTCTGGAATAGGCCACT |
|  |  | Reverse-AAGCCAGCTGTTCCAAGG |
| DCEF22 | DcWN_010436 | Forward-TGGAATGTGTGGCGATGA |
|  |  | Reverse-CAGACGTAAGTCCCATGGTT |
| DCEF23 | DcWN_013243 | Forward-AGAGGCTTTGCCAAGTACG |
|  |  | Reverse-GGAAACGCAAAATGTTAGCTC |
| DCEF26 | DcWN_027964 | Forward-GCACTAAGCCAAAAGTTAGCTC |
|  |  | Reverse-CAAATTGTGCTGGAAGTACGA |
| DCEF27 | DcWN_028357 | Forward-GTGCACACCAGGTTCTTC |
|  |  | Reverse-CCCTTTTGCCATTAAAGGAG |
| DCEF28 | XP_026685929.1 | Forward-CGGTCACGGACATGAACA |
|  |  | Reverse-CTTGGCATGTCCCAGATG |
| DCEF32 | XP_008477481.1 | Forward-GTAAGTTGGTTGCTGAATAAGGTG |
|  |  | Reverse-AACATCCCAGTCCACATTCC |
| DCEF33 | XP_008475409.1 | Forward-TTGGAGCTGTCTTCGTTTCTG |
|  |  | Reverse-CGTGTTCCCAACTGAAGAA |
| DCEF35 | XP_008468032.1 | Forward-ATGCATACACGTGTCTCCTAACA |
|  |  | Reverse-GGACTCTCCTCGGATTTCTT |
