# Supplementary material for: Gene silencing of Diaphorina citri candidate effectors promotes changes in feeding behaviors
Source: Sci Rep. 2020 Apr 7;10:5992. doi: 10.1038/s41598-020-62856-5 (PMC7138822; doi:10.1038/s41598-020-62856-5)
Supplement: Supplementary file 8 — supplementary information 8. [file 41598_2020_62856_MOESM8_ESM.docx]

**Gene silencing of *Diaphorina citri* candidate effectors promotes changes in feeding behaviors**

**Supplementary Information**

**Author affiliation:**

**Inaiara de Souza Pacheco**

Centro de Citricultura Sylvio Moreira, Instituto Agronômico de Campinas, Cordeirópolis, São Paulo, Brazil. Universidade Estadual de Campinas, Campinas, São Paulo, Brazil.

**Diogo Manzano Galdeano**

Centro de Citricultura Sylvio Moreira, Instituto Agronômico de Campinas, Cordeirópolis, São Paulo, Brazil.

**Nathalie Kristine Prado Maluta**

Instituto Agronômico de Campinas, Campinas, São Paulo, Brazil

**Joao Roberto Spotti Lopes**

Escola Superior de Agricultura “Luiz de Queiroz” - Universidade de São Paulo, Piracicaba, São Paulo, Brazil.

**Marcos Antonio Machado**

Centro de Citricultura Sylvio Moreira, Instituto Agronômico de Campinas, Cordeirópolis, São Paulo, Brazil.

**Corresponding author**

**Inaiara de Souza Pacheco**

Centro de Citricultura Sylvio Moreira, Instituto Agronômico de Campinas, Cordeirópolis, São Paulo, Brazil. Universidade Estadual de Campinas, Campinas, São Paulo, Brazil.

Email: inaiara@ccsm.br

**Supplementary Table S4:** Mean (± SE) non-sequential EPG variable values for the probing behaviors of *Diaphorina citri* on ‘Rangpur lime’ seedlings during an 8-h recording.

| Non-sequential  variables | Treatment | PPW^a^ | NWEI^a^ | *P^b^* | WDI^a^ | *P* | WDE^a^ | *P* |
| --- | --- | --- | --- | --- | --- | --- | --- | --- |
| np | **Sucrose** | 15/15 | 5.6 ± 1.1 a | 0.410 | 137.4 ± 19.4 a | 0.28 | 24.5 ± 3.9 ab | 0.01 |
|  | **GFP** | 15/15 | 3.2 ± 0.1 a |  | 91.9 ± 5.4 a |  | 28.7 ± 7.2 a |  |
|  | **DECF27** | 15/15 | 2.7 ± 0.4 a |  | 103.3 ± 26.2 a |  | 38.7 ± 8.4 bc |  |
|  | **DCEF28** | 15/15 | 3.5 ± 0.7 a |  | 91.8 ± 20.1 a |  | 26.5 ± 5.7 ab |  |
|  | **DCEF32** | 15/15 | 3.3 ± 0.5 a |  | 139.8 ± 26.8 a |  | 42.8 ± 7.6 c |  |
| C | **Sucrose** | 15/15 | 10.1 ± 1.4 a | 0.172 | 92.1 ± 11.6 a | 0.50 | 9.2 ± 0.9 a | <0.01 |
|  | **GFP** | 15/15 | 6.7 ± 0.3 a |  | 130.0 ± 5.3 a |  | 19.3 ± 2.9 b |  |
|  | **DECF27** | 15/15 | 7.1 ± 0.9 a |  | 126.4 ± 19.3 a |  | 17.9 ± 2.9 b |  |
|  | **DCEF28** | 15/15 | 6.7 ± 1.4 a |  | 95.8 ± 19.8 a |  | 14.2 ± 2.2 b |  |
|  | **DCEF32** | 15/15 | 6.3 ± 1.0 a |  | 119.9 ± 20.5 a |  | 19.1 ± 2.7 b |  |
| Probe | **Sucrose** | 15/15 | 5.6 ± 1.1 a | 0.318 | 342.6 ± 19.4 a | 0.28 | 61.2 ± 11.7 a | <0.01 |
|  | **GFP** | 15/15 | 3.1 ± 0.1 a |  | 388.1 ± 5.4 a |  | 126.6 ± 23.7 ab |  |
|  | **DECF27** | 15/15 | 2.5 ± 0.4 a |  | 376.7 ± 26.2 a |  | 154.0 ± 25.0 b |  |
|  | **DCEF28** | 15/15 | 3.4 ± 0.7 a |  | 388.2 ± 20.1 a |  | 114.2 ± 21.9 b |  |
|  | **DCEF32** | 15/15 | 3.1 ± 0.5 a |  | 340.2 ± 26.8 a |  | 110.9 ± 20.2 b |  |
| G | **Sucrose** | 9/15 | 0.7 ± 0.7 a | 0.10 | 31.7 ± 9.2 a | 0.03 | 47.5 ± 7.2 a | 0.54 |
|  | **GFP** | 6/15 | 0.5 ± 0.04 a |  | 34.1 ± 3.5 a |  | 73.1 ± 19.7 a |  |
|  | **DECF27** | 11/15 | 0.9 ± 0.9 a |  | 58.8 ± 17.4 ab |  | 63.0 ± 14.7 a |  |
|  | **DCEF28** | 13/15 | 0.9 ± 0.1 a |  | 100.4 ± 28.7 b |  | 115.9 ± 30.9 a |  |
|  | **DCEF32** | 12/15 | 1.0 ± 0.2 a |  | 104.4 ± 26.0 b |  | 104.4 ± 23.5 a |  |
| D | **Sucrose** | 15/15 | 4.7 ± 0.9 a | 0.14 | 1.9 ± 0.4 a | 0.14 | 0.4 ± 0.02 ab | 0.02 |
|  | **GFP** | 15/15 | 3.9 ± 0.2 a |  | 1.5 ± 0.1 a |  | 0.4 ± 0.02 a |  |
|  | **DECF27** | 13/15 | 4.2 ± 0.8 a |  | 2.2 ± 0.7 a |  | 0.5 ± 0.04 b |  |
|  | **DCEF28** | 12/15 | 3.1 ± 1.1 a |  | 1.2 ± 0.5 a |  | 0.4 ± 0.02 ab |  |
|  | **DCEF32** | 11/15 | 2.7 ± 0.7 a |  | 1.3 ± 0.4 a |  | 0.5 ± 0.05 ab |  |
| E1 | **Sucrose** | 15/15 | 4.0 ± 0.7 a | 0.22 | 4.7 ± 1.0 a | 0.14 | 1.2 ± 0.7 a | 0.07 |
|  | **GFP** | 15/15 | 3.5 ± 0.2 a |  | 3.0 ± 0.2 a |  | 0.9 ± 0.1 a |  |
|  | **DECF27** | 13/15 | 3.2 ± 0.7 a |  | 2.5 ± 0.6 a |  | 0.8 ± 0.1 a |  |
|  | **DCEF28** | 12/15 | 3.0 ± 1.02 a |  | 3.6 ± 1.9 a |  | 1.2 ± 0.1 a |  |
|  | **DCEF32** | 11/15 | 2.1 ± 0.5 a |  | 2.7 ± 0.9 a |  | 1.3 ± 0.3 a |  |
| E2 | **Sucrose** | 15/15 | 1.9 ± 0.2 a | 0.09 | 212.2 ± 37.8 a | 0.184 | 109.8 ± 21.5 a | 0.97 |
|  | **GFP** | 15/15 | 1.7 ± 0.1 a |  | 219.4 ± 9.3 a |  | 131.6 ± 29.3 a |  |
|  | **DECF27** | 12/15 | 1.5 ± 0.3 a |  | 186.9 ± 38.4 a |  | 121.9 ± 25.6 a |  |
|  | **DCEF28** | 12/15 | 1.6 ± 0.3 a |  | 187.2 ± 38.7 a |  | 116.0 ± 25.5 a |  |
|  | **DCEF32** | 8/15 | 1.0 ± 0.4 a |  | 111.9 ± 39.2 a |  | 111.9 ± 9.0 a |  |
| E2s | **Sucrose** | 15/15 | 1.3 ± 0.1 a | 0.04 | - | - | 157.7 ± 23.9 a | 0.91 |
|  | **GFP** | 15/15 | 1.2 ± 0.0 a |  | - |  | 182.1 ± 33.9 a |  |
|  | **DECF27** | 12/15 | 1.1 ± 0.2 a |  | - |  | 163.6 ± 28.3 a |  |
|  | **DCEF28** | 12/15 | 1.3 ± 0.2 a |  | - |  | 146.9 ± 28.5 a |  |
|  | **DCEF32** | 7/15 | 0.6 ± 0.2 b |  | - |  | 184.5 ± 43.5 a |  |

**^a^** **PPW**, proportion of individuals that produced the waveform type; **NWEI,** number of waveform events per insect; **WDI**, Waveform duration (min) per insect; **WDE,** Waveform duration (min) per event; **^b^** Statistical comparisons between the treatments ‘Sucrose, GFP, DECF_27, DCEF28 and DCEF32’ or each parameter were made by: Tukey test (for Gaussian distribution variables) or non-parametric Kruskal-Wallis test (for non-Gaussian distribution variables). Underline-type indicates significant differences (P<0.05).
